# Matrix Metalloproteinase 9/microRNA-145 Ratio: Bridging Genomic and Immunological Variabilities in Thyroid Cancer

**DOI:** 10.3390/biomedicines11112953

**Published:** 2023-11-01

**Authors:** Eman A. Toraih, Mohamed H. Hussein, Essam Al Ageeli, Mohamad Ellaban, Shahd W. Kattan, Krzysztof Moroz, Manal S. Fawzy, Emad Kandil

**Affiliations:** 1 Division of Endocrine and Oncologic Surgery, Department of Surgery, School of Medicine, Tulane University, New Orleans, LA 70112, USA; mhussein1@tulane.edu (M.H.H.); ekandil@tulane.edu (E.K.); 2 Genetics Unit, Department of Histology and Cell Biology, Faculty of Medicine, Suez Canal University, Ismailia 41522, Egypt; 3 Department of Clinical Biochemistry (Medical Genetics), Faculty of Medicine, Jazan University, Jazan 45142, Saudi Arabia; ealageeli@jazanu.edu.sa; 4 Faculty of Medicine, Port Said University, Port Said 42526, Egypt; dr.labban@gmail.com; 5 Department of Medical Laboratory, College of Applied Medical Sciences, Taibah University, Yanbu 46411, Saudi Arabia; skattan@taibahu.edu.sa; 6 Department of Pathology and Laboratory Medicine, School of Medicine, Tulane University, New Orleans, LA 70112, USA; kmoroz@tulane.edu; 7 Department of Medical Biochemistry and Molecular Biology, Faculty of Medicine, Suez Canal University, Ismailia 41522, Egypt; manal2_khashana@ymail.com; 8 Department of Biochemistry, Faculty of Medicine, Northern Border University, Arar 91431, Saudi Arabia

**Keywords:** MMP9/miR-145 ratio, biomarker, thyroid cancer, PTC, *BRAF*, perioperative assessment

## Abstract

Matrix metalloproteinase 9 (MMP9) and microRNA-145 (miR-145) have emerged as essential biomarkers in thyroid cancer progression and metastasis. However, their combined evaluation and clinical utility as a unified prognostic marker across diverse thyroid cancer subgroups remain unexplored. We investigated the diagnostic and prognostic value of the MMP9/miR-145 ratio in thyroid cancer, hypothesizing it may overcome inter-patient heterogeneity and serve as a versatile biomarker regardless of genetic mutations or autoimmune status. MMP9 and miR-145 expressions were analyzed in 175 paired papillary thyroid cancer (PTC) and normal tissues. Plasma levels were assessed perioperatively and longitudinally over 12–18 months in 86 matched PTC patients. The associations with clinicopathological parameters and patient outcomes were evaluated. MMP9 was upregulated, and miR-145 downregulated in cancer tissues, with a median MMP9/miR-145 ratio 17.6-fold higher versus controls. The tissue ratio accurately diagnosed thyroid malignancy regardless of *BRAF* mutation or Hashimoto’s thyroiditis status, overcoming genetic and autoimmune heterogeneity. A high preoperative circulating ratio predicted aggressive disease features, including lymph node metastasis, extrathyroidal extension, progression/relapse, and recurrence. Although the preoperative plasma ratio was elevated in patients with unfavorable outcomes, it had limited utility for post-surgical monitoring. In conclusion, the MMP9/miR-145 ratio is a promising biomarker in PTC that bridges genetic and immunological variabilities, enhancing preoperative diagnosis and prognostication across diverse patient subgroups. It accurately stratifies heterogenous cases by aggressiveness. The longitudinal trends indicate decreasing applicability for post-thyroidectomy surveillance. Further large-scale validation and protocol standardization can facilitate clinical translation of the MMP9/miR-145 ratio to guide personalized thyroid cancer management.

## 1. Introduction

Thyroid cancer (TC) represents a significant global health burden due to its rising incidence over the past several decades [1]. This disease not only places a substantial physiological burden on patients but also incurs significant socioeconomic costs due to long-term management and treatment-related complications [2]. Despite its growing prevalence, effective diagnosis, prognosis, and treatment markers are urgently needed to enhance clinical decision-making and overcome associated challenges [3]. TC presents numerous complexities, not only in its pathogenesis and clinical presentation but also in the diversity of patients it affects. Factors such as *BRAF* mutations and conditions like autoimmune Hashimoto’s thyroiditis can significantly influence the disease’s progression and the patient’s response to treatment [4,5]. *BRAF* mutations, particularly *BRAF*^V600E^, are the most common genetic alterations in papillary thyroid cancer (PTC), the most prevalent type of thyroid cancer. They have been associated with aggressive tumor behavior and poor clinical outcomes [6,7]. On the other hand, Hashimoto’s disease, an autoimmune disorder, is the most common cause of hypothyroidism and has been linked to an increased risk of TC and less aggressive features [4,8]. Consequently, developing a “one test fits all” strategy to diagnose and predict the course of TC can be challenging.

Our previous research identified miR-145 as a promising marker for diagnosing and prognosticating TC, with potential therapeutic applications [9]. The dysregulation of miR-145 has been linked to essential clinicopathological features of TC, including tumor size, lymph node metastasis, and recurrence [10,11]. Through in silico data analysis, we discovered that miR-145 negatively regulates multiple genes in the TC signaling pathway [KEGG: hsa05216], affecting critical processes such as cell apoptosis, proliferation, stem cell differentiation, angiogenesis, and metastasis [12,13,14,15].

Significantly, an analysis of RNA-seq data from The Cancer Genome Atlas (TCGA) brought our attention to matrix metalloproteinase-9 (*MMP9*), a zinc-dependent endopeptidase that emerged as one of the upregulated protein-coding genes in recurrent thyroid cancer samples. MMP9, a major protease in extracellular matrix (ECM) degradation and leukocyte migration, is one of the targets of miR-145, with its expression significantly reducing miR-145-expressing cells in other cancer types [16]. This observation prompted us to investigate the potential significance of the MMP9/miR-145 ratio in TC progression, specifically in the context of *BRAF* mutations and Hashimoto’s disease. MMP9 plays a multifaceted role in influencing ECM remodeling, angiogenesis, and metastasis, among other mechanisms [17,18,19]. These actions contribute to disease progression, increased aggressiveness, and resistance to therapy [19,20]. Its overexpression has been frequently observed in cancer, suggesting its association with invasiveness [21]. Additionally, the interaction of MMP9 with other signaling pathways and its involvement in the tumor microenvironment further complicates its role in TC [20,22,23].

Given the intricate nature of MMP9 and miR-145 and its potential relevance in TC, evaluating the MMP9/miR-145 ratio in patients with *BRAF* mutations and Hashimoto’s disease holds immense potential for gaining valuable insights. This ratio has the potential to shed light on the molecular mechanisms driving these conditions, predict disease progression, and guide the selection of appropriate therapeutic strategies. By investigating the robustness of the MMP9/miR-145 ratio across these diverse patient groups, we aim to develop a versatile, all-encompassing diagnostic and prognostic tool for TC. Ultimately, we aim to establish the MMP9/miR-145 ratio as a universal marker that transcends the genetic and immunological heterogeneity observed in TC patients. Achieving this goal will streamline TC diagnosis, prognostication, and treatment planning by providing a unified and efficient testing solution.

## 2. Materials and Methods

### 2.1. Analysis of Tissues and Blood Specimens of Thyroid Cancer Patients

#### 2.1.1. Study Population

The study population consisted of patients diagnosed with thyroid cancer who underwent thyroidectomy. A total of 350 tissue samples from 175 patients were retrospectively collected from the tumor biobanks of Tulane University, LA, USA and El bayan Pathology Laboratory, Port-Said, Egypt between January 2010 and December 2015. This study focused on adult cohorts aged 20 years or older diagnosed with PTC according to the International Classification of Oncological Diseases, 4th edition. Prior to operative resection, the patients did not receive any treatment. The exclusion criteria involved follicular, Hürthle cell, poorly differentiated, anaplastic, and medullary TC. Patients with incomplete follow-up, missing data, or unpaired adjacent samples were also excluded. This study adhered to the guidelines of the Declaration of Helsinki and we obtained approval from the Institutional Review Board of Tulane University, USA (protocol code 2020-1636, 08/12/2020), and the Ethical Committee of Suez Canal University, Egypt (protocol code 4344, 03/11/2020). Since this study utilized archived formalin-fixed paraffin-embedded (FFPE) and blood specimens, patient consent was waived. The patient data obtained from hospital medical records were anonymized and de-identified prior to analysis.

#### 2.1.2. Clinical and Pathological Assessment

Clinical and pathological data were obtained from patient records for comprehensive evaluation. The collected data encompassed various aspects including demographic variables such as age at diagnosis, sex, and year of diagnosis. Additionally, comorbidities such as obesity, HCV infection, Hashimoto’s disease, and smoking history were documented. The results of *BRAF* mutation screening were retrieved from patient records. The disease characteristics were assessed, including TNM stage according to the 8th edition, presence, or absence of multifocal disease, minor or gross extrathyroidal extension, and lymphovascular invasion. Information regarding treatment strategies, response to therapy, recurrence, and mortality was also recorded. Several clinical endpoints were defined for the study. These endpoints included: (a) Relapse-free survival (RFS): this denotes the time from treatment initiation to the occurrence of relapse, which can be local, regional, or distant. (b) Disease-free survival (DFS): this represents the duration from treatment initiation to the development of locoregional recurrence, metastasis, or death (for any reason). (c) Overall survival (OS): this signifies the period from diagnosis or treatment initiation to death (for any reason) [9]. Clinical recurrence was defined as the reappearance of pathologically confirmed malignant tissue, the emergence of metastatic lesions in organs such as the lungs, bone, and brain, and/or the presence of biochemical and/or new structural evidence of disease in patients who were initially rendered disease-free. Persistent disease referred to residual tumors in the area of the primary tumor and/or regional lymph nodes after the initial surgery. No clinical evidence of disease (NCED) was determined based on the absence of disease, as assessed through physical examination, neck ultrasonography, neck computed tomography (CT) scans, and other relevant imaging techniques conducted during clinical evaluation at the end of the follow-up period, regardless of serum thyroglobulin concentration. For individuals without tumor relapse, the last follow-up time without a recurrence event was used for analysis.

#### 2.1.3. Tissue Sample Processing and Histopathological Assessment

Archived FFPE tissues were collected. A total of 175 paired samples of thyroid cancer and non-tumor thyroid tissues were included in the study. The histopathological diagnosis PTC was confirmed by two independent pathologists, who also assessed the subtype variant and staging. To ensure precise identification of cancer and control tissues within the FFPE specimens, laser microdissection was performed using a Leica PBI Laser Microdissection. The tissues were cut into 4 µm serial sections and stored at 4 °C until further use. For histological evaluation, a 4 µm thick section was subjected to hematoxylin and eosin (H&E) staining. Additionally, 3–4 sections were extracted from each sample and stored in Eppendorf tubes for subsequent qRT-PCR experiments.

#### 2.1.4. Blood Sampling Procedure and Longitudinal Follow-Up

Prior to surgical intervention, blood samples were collected from 86 patients. This initial preoperative sampling was followed by an immediate postoperative sampling within 3–5 days after tumor resection. The resected specimens from these surgeries were meticulously examined for the presence of lymph node metastasis (LNM) and any signs of extrathyroidal extension (ETE). Six months post-surgery, out of the initial cohort, 69 patients underwent another round of blood sampling. These patients were simultaneously assessed for any radiological or clinically evident LNM or any signs of recurrence. A final blood sampling was conducted on 48 of these patients between 12 and 18 months post-surgery to monitor for LNM, distant metastasis, or recurrence. For the blood sampling procedure, peripheral venous blood was drawn from cubital fossa into a vacutainer containing EDTA. Upon collection, the samples were promptly centrifuged within an hour to separate the plasma. The plasma was then aliquoted and stored at a temperature of −80 °C. The samples remained under these conditions for a duration between 18 and 22 months. Following this storage period, 287 specimens (86 preoperative, 86 immediate postoperative, 69 samples from 6 months post-operation, and 48 from 12 months post-operation) underwent a single thawing process before being processed for transcriptomic analysis.

#### 2.1.5. Total RNA/miRNA Extraction

Total RNA, encompassing both large and small RNA molecules, was extracted from FFPE samples using a combination of xylene deparaffinization and the Qiagen miRNeasy FFPE Isolation kit (Qiagen, Hilden, Germany; Catalog number 217504). First, total RNA including miRNA was extracted from blood using the Qiagen miRNeasy Micro Kit (Qiagen, Hilden, Germany; Catalog number 217084), following the manufacturer’s instructions. The extracted RNA samples were then assessed for concentration and purity using a Nanodrop ND-1000 spectrophotometer (NanoDrop Tech. Inc., Wilmington, DE, USA), utilizing a wavelength-dependent extinction coefficient of 33 [24]. To maintain RNA integrity, the samples were stored in separate aliquots at a temperature of −80 °C until further analysis.

#### 2.1.6. miR-145 Expression Analysis

A total amount of 10 ng RNA was initially transcribed into complementary DNA (cDNA) using the TaqMan miRNA Reverse Transcription (RT) kit (P/N 4366596; Thermo Fisher, Applied Biosystems, Foster City, CA, USA) on a T-Professional Basic, Biometra PCR system (Biometra, Goettingen, Germany) under the pre-defined amplification parameters of 16 °C for 30 min and 42 °C for the same duration, followed by 85 °C for 5 min, with a final holding at 4 °C. For normalization purposes, we utilized 5× stem-loop primers specific to miR-145 or the endogenous control separately. The successful removal of DNA contaminants was validated via no-reverse transcription controls from selected samples. We adhered to the “Minimum Information for Publication of Quantitative Real-time PCR Experiments (MIQE)” guidelines for conducting qRT-PCR experiments [25]. The quantification of the assigned miRNA incorporated a final reaction volume of 20 µL, composed of 1.33 µL RT product of the selected miRNA, 2× TaqMan Universal PCR master mix, including the “Uracyl N-Glycosylas (UNG)” (Applied Biosystems, P/N 4440043) to prevent carry-over contamination of dU-containing DNA from previous reactions, and 1 µL 20× TaqMan small RNA assay (hsa-miR-145; assay ID: 002278 with mature miRNA sequence:GUCCAGUUUUCCCAGGAAUCCCU) or small nuclear RNA U6 (RNU6B; assay ID: 001093 with (CGCAAGGATGACACGCAAATTCGTGAAGCGTTCCATATTTTT) sequence). As additive internal control assays to secure data normalization, three other tests—specifically RNU48, let-7a, and miR-16—were included. This was in line with recent guidelines that emphasize using quantitative reverse-transcription polymerase chain reaction (qRT-PCR) for miRNA study and endogenous control evaluation in archived PTC specimens [26]. Owing to the uniformity and reliability of RNU6B across various samples, it was employed to normalize data. The PCR procedure was conducted using the StepOne Real-time PCR system (manufactured by Applied Biosystems). The incubation stages were an initial 95 °C for 10 min, followed by 45 cycles consisting of 15 s at 92 °C and 1 min at 60 °C. The reactions were executed in triplicate, with any standard deviation exceeding 2.0 tagged as an outlier. Suitable controls were an integral part of each experimental run [27].

#### 2.1.7. MMP9 mRNA Expression Analysis

As previously mentioned, for the execution of qRT-PCR procedures, we adhered meticulously to the Minimum Information for Publication of Quantitative Real-Time PCR Experiments (MIQE) guidelines [25]. The first step of the amplification process was the reverse transcription (RT) of 1 µg of total RNA to yield cDNA using a High-Capacity cDNA RT Kit (P/N 4368814, Applied Biosystems, Foster City, CA, USA). The second step was real-time PCR reaction using a specific TaqMan probe for the *MMP9* (Assay no. Hs00957562_m1), compared to the housekeeping gene glyceraldehyde 3-phosphate dehydrogenase (*GAPDH*) for normalization of the data.

In each run, appropriate negative controls were applied: no template and no reverse transcriptase controls. The final volume of the reaction (20 µL) contained RT products (1.33 µL), 2× TaqMan Universal PCR Master Mix (10 µL), and TaqMan RNA assay (1 µL). The StepOne Real-Time PCR was set up as follows: 10 min at 95 °C, followed by 40 cycles of 15 s at 92 °C and 60 s at 60 °C. The cycle of quantification (Cq) refers to the cycle count when the fluorescence surpasses a set threshold level [25]. The calculation of the relative quantity of *MMP9* to *GAPDH* transcripts in patients versus controls was performed using the formula “2^−ΔΔCq^; where ΔΔC_q_  =  (C_q_ *MMP9* − C_q_ *GAPDH*)_TC_ − (C_q_ *MMP9* −  C_q_ *GAPDH*)_mean controls_” [28]. Similar calculation steps were performed for miRNA and *RNU6B* expression.

#### 2.1.8. Tissue Microarray (TMA)

Consecutive sections of two tissue microarrays (TMAs) were obtained from TissueArray.Com LLC. (Derwood, MD, USA) for protein analysis and mutation screening of *BRAF*. The TMAs (TH808 and TH8010a) included TC and normal thyroid tissues. The TH808 TMA comprised 27 cases of PTC, 3 cases of follicular adenocarcinoma, and 5 cases each of adjacent normal tissue and normal tissue. This TMA had duplicate cores per case, totaling 80 cores across 40 cases. The TH8010a TMA consisted of 80 cases with different stages: 27 cases in stage I, 17 cases in stage II, 19 cases in stage III, and 7 cases in stage IV. Additionally, it contained 10 normal tissue samples, with a single core per case. All tissue samples in the TMAs had a diameter of 1.5 mm, allowing for efficient analysis and comparison of multiple samples within a single microarray.

#### 2.1.9. Immunofluorescence Analysis and Quantification of Protein Expression Data

We carried out an immunofluorescence study on 5 µm thick thyroid tissue sections mounted on Superfrost Plus Microscope slides to evaluate MMP9 protein and CD45+ immune cell expression and localization. After baking the slides at 60 °C for 2 h, they were deparaffinized in xylene, rehydrated through graded ethanol solutions, and immersed in double-distilled water. Epitope retrieval involved microwaving the slides in a Tris-based solution (pH 9) with 0.01% Tween 20 for 16 min, followed by cooling in a citrate-based solution (pH 6.0). The tissue sections were then washed with a phosphate-buffered saline and fish gelatin mix (PBS-FSG) before being placed in a humid chamber. To block non-specific interactions, they were treated with 1% normal donkey serum (NDS) for 40 min. The sections were subsequently incubated with primary antibodies, detailed in Supplementary Table S1, for 60 min, followed by Alexa Fluor-conjugated secondary antibodies: Alexa Fluor 488 Donkey anti-Goat (diluted 1:1000 in NDS) for MMP9 and Alexa Fluor 555 Donkey anti-Mouse (diluted 1:50) for CD45, each for 40 min. After these treatments, the nuclei were stained with 4’,6-diamidino-2-phenylindole (DAPI), and the slides were sealed using a custom anti-quenching medium containing Mowiol and DABCO. The immunofluorescence images were acquired with a Zeiss Axio Scan. Z1 Slide Scanner (Zeiss, Oberkochen, Germany).

The protein expression data for MMP9 were determined using a semi-quantitative method that combined both the intensity and extent of staining. All samples were examined independently and blindly by two pathologists who were uninformed about the clinical details and objectives of the study, thereby ensuring objective assessment and minimizing potential bias. Autofluorescence was assessed and excluded during the quantification of protein markers. The intensity of MMP9 and CD45 marker staining was assessed using a four-point scale: 0 (none), 1 (weak), 2 (moderate), and 3 (strong). Additionally, the proportion of positively stained areas divided by DAPI area was categorized into quartiles to one of four categories (1 to 4) according to expression quartiles. The final score for each sample was computed by multiplying the intensity score (0–3) with the percentage score (1–4), yielding a range from 0 to 12.

### 2.2. In Silico Data Analysis

The genomic structure of the MMP9 protein was accessed from databases such as the Ensembl human genome browser (https://useast.ensembl.org/index.html) and the UniProt KnowledgeBase (https://www.uniprot.org). These databases provide detailed information on the genomic and protein structure of MMP9, respectively. To analyze the interactions between microRNAs and their target genes, the miRTarBase database was utilized (https://mirtarbase.cuhk.edu.cn/~miRTarBase/miRTarBase_2022/php/index.php). This database compiles experimentally validated microRNA-target interactions (MTIs), which are confirmed through various experimental methods including reporter assay, Western blot, microarray, and next-generation sequencing experiments. We specifically looked at predicted complementary sites at the 3′untranslated region of MMP9 using miRanda v3.3, a miRNA target scanner that aims to predict mRNA targets for microRNAs using dynamic programming alignment and thermodynamics (https://cbio.mskcc.org/miRNA2003/miranda.html). Additionally, the correlation between miR-145 and MMP9 was investigated using multiple datasets from the Gene Expression Omnibus (GEO) database (https://www.ncbi.nlm.nih.gov/geo/) (all the previously mentioned URL were last accessed on 6 September 2023). GEO is a public functional genomics data repository and is an essential resource for analyzing gene expression patterns and alterations. Ingenuity Pathway Analysis (IPA) version 01-22-01 was utilized to perform pathway explorer analysis connecting miR-145, MMP9, and thyroid cancer using millions of knowledgebase databases in the literature. Human experimentally based studies were selected.

### 2.3. Systematic Review on the Clinical Significance of MMP9 and miR-145

A systematic search was conducted in October 2022 across PubMed, ScienceDirect, Web of Science, and Google Scholar using the following keywords: (“thyroid”) AND (“malignancy” or “neoplasms” or “tumor” or “carcinomas” or “cancers”) and (“matrix metalloproteinase 9” or “gelatinase B” or “Type IV Collagenase” or “MMP9”). There were no restrictions on the time of publication or language. The studies eligible for inclusion involved tissue or blood samples from patients diagnosed with PTC, with preoperative or postoperative data on MMP9 mRNA or protein expression levels in tumor versus non-tumor samples. Exclusion criteria comprised non-human studies (in vivo or in vitro), case reports/series, conference abstracts, reviews, or editorial letters. The objective was to identify relevant articles investigating the association between MMP9 and any clinical or pathological characteristics such as lymph node metastasis, extrathyroidal extension, capsular invasion, poor survival, TNM staging, and tumor size. Search and data extraction were performed by two authors and reviewed by a third person. Following the removal of duplicates, titles and abstracts were screened, and full-text articles were assessed. The data extracted included the first author, year, geographic region, study period, analysis method, sample source (FFPE, fresh, serum, plasma, fine needle aspirate), sample type (RNA/protein), sample size (tumor/non-tumor), expression levels, and associations with clinical parameters. These data were compiled into a predesigned Excel sheet. For the meta-analysis, a one-arm meta-analysis and pairwise comparison were used to aggregate the estimated expression levels of the MMP9 marker in tumor versus non-tumor samples. The mean difference was reported if data from both arms were available. A fixed-effects model was employed unless there was significant heterogeneity (I^2^ > 50%), in which case a random-effects model was used instead.

### 2.4. Statistical Analysis

The statistical analysis in this study was conducted using RStudio and SPSS v28.0. Given the non-parametric distribution of biomarker expressions in cancerous versus normal tissues, different non-parametric tests were utilized. For paired two-group comparisons, the related-samples Wilcoxon signed rank test was used, whereas Friedman’s two-way ANOVA was implemented for multiple group comparisons. On the other hand, for unpaired groups, the Mann–Whitney U test was applied for two-sample comparisons, and the Kruskal–Wallis one-way ANOVA was used for comparisons involving more than two samples. Additionally, plasma expression levels at various time points were assessed using related-samples Friedman’s two-way analysis of variance by ranks, with Bonferroni correction applied to adjust for multiple testing. A Spearman’s correlation analysis was employed to determine the relationship between the expression levels of biomarkers in blood and tissues. The diagnostic accuracy of the biomarkers was evaluated using receiver operator characteristics (ROC) curve analysis, with the area under the curve (AUC) serving as a measure to compare the performance of different biomarkers. All analyses were two-tailed, and a *p*-value of less than 0.05 was considered statistically significant. Logistic binary regression was used to predict the risk factors associated with lymph node metastasis and the odds ratio (OR) and 95% confidence interval were reported. Meanwhile, the Cox hazard proportional regression was used to identify the risk factors associated with disease progression and relapse and the hazards ratio (HR) and 95% confidence interval were reported. The MMP9/miR-145 ratio was utilized to classify patients into high expressors and low expressors using a cutoff value of 15. Kaplan–Meier survival curves were generated to assess relapse-free survival times, disease-free survival times, and overall survival. The log rank (Mantel–Cox) test was used for comparison.

## 3. Results

### 3.1. Analysis of Tissues and Blood Specimens of Thyroid Cancer Patients

#### 3.1.1. Characteristics of the Study Population of Tissue Samples

This study investigated a cohort of 175 thyroid cancer patients and provided a comprehensive overview of their demographic, genomic, pathological, and clinical characteristics. In our cohort, a significant number of individuals were under 55 years of age (*n* = 119, 68%), with females accounting for a notable proportion of the total (*n* = 119, 68%). Notably, most of the study population was obese (BMI ≥ 30 kg/m^2^, *n* = 161, 92%), and only 15 patients reported being smokers (8.5%). Furthermore, the cohort also encompassed a considerable number of individuals with a positive diagnosis for hepatitis C virus (*n* = 76, 43.4%). Pathological evaluations revealed that most tumors were unilateral (*n* = 120, 68.6%), unifocal (*n* = 145, 82.9%), and mostly detected at early T stages (T1a or T1b, *n* = 93, 53.1%). Nodal metastasis was present in 74 cases accounting for 42.2%, 30 patients (17.1%) presented with distant metastasis, and many patients were classified as stage IA (*n* = 115, 65.7%). Extrathyroidal extension and lymphovascular invasion were identified in 21.7% (*n* = 38) and 13.7% (*n* = 24) of the patients, respectively. RAS gene mutations were less frequent, seen in only 8.6% (*n* = 15) of the studied individuals. Regarding treatment modalities, a considerable number of patients underwent a total/subtotal thyroidectomy (*n* = 99, 56.6%) and neck dissection (*n* = 94, 53.7%). Radioactive iodine (RAI) therapy, utilized as an adjuvant treatment to eliminate any remnant thyroid tissue post-thyroidectomy, was administered in 30.9% of patients (*n* = 54). Additionally, external beam radiation therapy (EBRT) was used in the treatment of more than half of the patient cohort (*n* = 91, 52.0%). In terms of disease evolution, 42.3% (*n* = 74) of the patients experienced disease progression or relapse, and 17.7% (*n* = 31) had disease recurrence. Despite these figures, the mortality rate remained comparatively low, with only 4.6% (*n* = 8) of the patients having died due to the disease. Overall, the cohort comprised both *BRAF*^V600E^ mutation-positive (*n* = 78, 44.6%) and wild-type (*n* = 97, 55.4%) patients, as well as individuals with Hashimoto’s thyroiditis (*n* = 26, 14.9%) and without this condition (*n* = 149, 85.1%). Comparative analysis of these groups is outlined in Table 1. In our study, patients harboring the *BRAF*^V600E^ mutation displayed a higher incidence of multifocal lesions (*p* = 0.027), extranodal extension (*p* = 0.036), distant metastasis (*p* < 0.001), and advanced stage IV (*p* = 0.027). Furthermore, those with Hashimoto’s thyroiditis exhibited a 26.9% prevalence of RAS mutations, compared to only 5.4% in those without the condition (*p* = 0.002).

#### 3.1.2. Diagnostic Performance of MMP9/miR-145 Ratio in Thyroid Tissues

**Figure 1:**
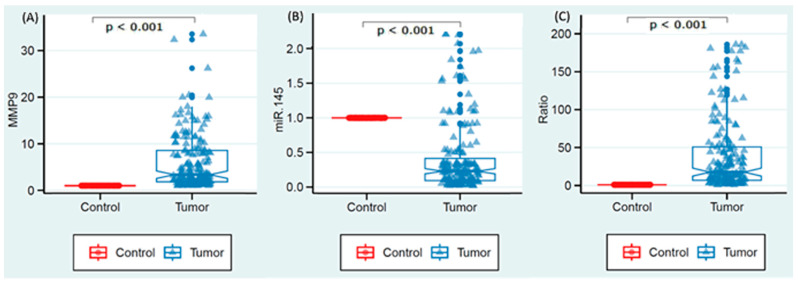
MMP9 and miR-145 expression pattern in thyroid tumors compared to normal controls. (**A**) The expression level of MMP9 RNA in tissues. (**B**) The expression level of miR-145 in tissues. (**C**) The ratio between the expression levels of MMP9 and miR-145 in thyroid tissues. The box represents the middle 50% of the data (between 25th and 75th percentiles). The whiskers indicate the ranges for the upper and lower 25% of the data, excluding outliers. The triangles represent individual data points for each tumor sample. The dots outside the whiskers represent the mean values of the outliers at that height. Paired non-tumor and tumor samples (*n* = 175 patients) were compared. Related-samples Wilcoxon signed rank test was used. Statistical significance was considered at *p* < 0.05.

In the current investigation, paired tissue samples (175 cancerous and 175 non-cancerous) were assessed for the expression levels of MMP9 and miR-145 to evaluate their diagnostic potential in thyroid tumors. As depicted in Figure 1, MMP9 exhibited a marked upregulation in the cancerous tissues, with a median expression level of 3.35 (IQR = 1.83–8.55), *p* < 0.001. All cases showed an upregulation level greater than 1.0, indicating a consistent pattern of elevated MMP9 expression in thyroid cancer tissues compared to their non-cancerous counterparts. In contrast, miR-145 demonstrated a significantly lower expression level in the thyroid tumors, with a median relative expression of 0.23 (IQR = 0.10–0.42) compared to controls of an expression set at 1.0, *p* < 0.001. This under-expression was observed in the majority of cases, with 153 (87.4%) showcasing downregulation. However, 22 cases (12.6%) deviated from this pattern, demonstrating over-expression values greater than 1.0. The ratio of MMP9 to miR-145 expression levels in the cancerous tissues further highlighted this inverse relationship, exhibiting a median of 17.62 (IQR = 6.81–50.85, *p* < 0.001). Figure 1 illustrates the expression pattern of MMP9 and miR-145 in thyroid tumors in comparison to normal controls. Panel A represents the expression level of MMP9 RNA in tissues, Panel B displays the expression level of miR-145 in tissues, and Panel C demonstrates the ratio between the expression levels of MMP9 and miR-145 in thyroid tissues.

Table 2 shows a comparison between cohorts with lower and higher expression of miR-145 in tumor tissues compared to paired normal tissues. The data demonstrate that cohorts with *BRAF* mutation were more likely to have upregulated miR-145 levels (68.2% vs. 41.2%, *p* = 0.022), while those with HCV were less likely to exhibit high expression pattern (4.5% vs. 49%, *p* < 0.001). An ROC curve analysis demonstrated high diagnostic accuracy of MMP9/miR-145 ratio at cutoff 1.14 with an AUC of 0.989 ± 0.008 (*p* < 0.001) and sensitivity of 98.9% (95% CI = 95.9–99.8%). The accuracy yielded better sensitivity at higher cutoff level in the presence of Hashimoto’s thyroiditis (cutoff: 1.45) and HCV infection (cutoff: 1.64).

#### 3.1.3. Prognostic Performance of MMP9/miR-145 Ratio

Table 3 provides a comprehensive examination of the association between MMP9/miR-145 expression and clinical-pathological characteristics of thyroid tumors across 175 cases. The data are stratified by various clinical, genomic, and pathological parameters for MMP9, miR-145, and their ratio (MMP9/miR-145). Those positive for HCV had increased MMP9 levels, reduced miR-145 levels, and a higher MMP9/miR-145 ratio compared to HCV-negative individuals (*p* < 0.001). Patients with nodal infiltration, distant metastasis, progression, relapse, or recurrence exhibited higher MMP9 values and MMP9/miR-145 ratios compared to their respective counterparts without these clinical attributes. These patterns suggest that MMP9 and the MMP9/miR-145 ratio might serve as potential biomarkers for aggressive disease features and outcomes.

**Figure 2:**
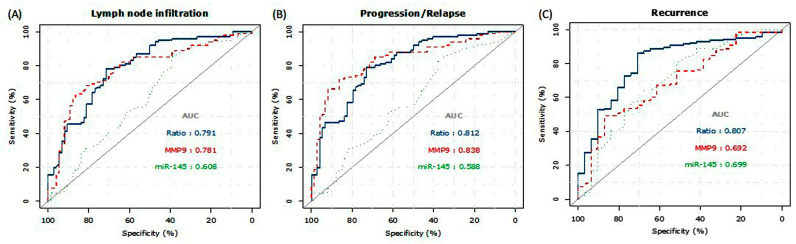
Prognostic accuracy of MMP9 and miR-145 expression in papillary thyroid carcinoma. Receiver operator characteristics (ROC) curve analysis was employed for MMP9, miR-145, and the ratio MMP9/miR-145. Areas under the curve (AUCs) were estimated and compared for detecting (**A**) lymph nodal metastasis. (**B**) Disease progression and relapse. (**C**) Recurrence. Statistical significance was considered at *p* < 0.05. The AUC is a pivotal metric that quantifies the diagnostic capability of a biomarker or test to differentiate between the positive and negative groups. An AUC of 1.0 signifies perfect discrimination, whereas an AUC of 0.5 indicates a performance no better than chance.

The ROC analysis evaluated the prognostic efficacy of three indicators: MMP9, miR-145, and the MMP9/miR-145 ratio (Figure 2). The MMP9/miR-145 ratio and MMP9 emerged as strong predictors for LNM with respective AUCs of 0.791 (95% CI: 0.724–0.859, *p* < 0.001) and 0.781 (95% CI: 0.711–0.852, *p* < 0.001). In contrast, miR-145 demonstrated a moderate impact, having an AUC of 0.608 (95% CI: 0.522–0.696, *p* = 0.014). In predicting progression and relapse, the ratio and MMP9 were substantial, achieving AUCs of 0.812 (95% CI: 0.749–0.876, *p* < 0.001) and 0.838 (95% CI: 0.778–0.898, *p* < 0.001), respectively. miR-145 lagged with an AUC of 0.589 (95% CI: 0.502–0.675, *p* = 0.045). For recurrence predictions, the ratio led with an AUC of 0.807 (95% CI: 0.721–0.893, *p* < 0.001), while MMP9 and miR-145 had AUCs of 0.692 (95% CI: 0.593–0.792, *p* = 0.001) and 0.699 (95% CI: 0.590–0.808, *p* = 0.001), respectively. Given its efficacy, the MMP9/miR-145 ratio was chosen for subsequent analyses.

#### 3.1.4. Stratified Analysis

In stratified analysis considering both *BRAF* mutation status and Hashimoto thyroiditis, Table 4 elucidates the prognostic utility of the MMP9/miR-145 ratio across critical clinical endpoints: nodal metastasis, progression/relapse, and recurrence. For nodal metastasis, individuals without the *BRAF* mutation (*BRAF*−) demonstrate a robust predictive power with an AUC of 0.854, sensitivity at 86.0%, and specificity at 70.4%. In contrast, the *BRAF*+ cohort shows a diminished AUC of 0.722, a reduced sensitivity of 54.8%, yet a slightly better specificity of 74.5%. Notably, when considering Hashimoto’s thyroiditis, those without the condition (HT-) have an AUC of 0.798 with balanced sensitivity (72.7%) and specificity (72.3%). However, the HT+ subset showcases an AUC of 0.757 with comparable sensitivity (75.0%) and specificity (72.2%). Regarding progression/relapse, the *BRAF*− subset excels with an AUC of 0.892, high sensitivity of 90.2%, and specificity of 71.4%. In contrast, *BRAF*+ individuals attain an AUC of 0.733, a moderate sensitivity of 54.5%, and specificity of 75.6%. The HT- cohort offers an AUC of 0.797, closely matched sensitivity (72.3%), and specificity (71.4%). Those with HT present a high AUC of 0.895, sensitivity (88.9%), and specificity (82.4%). In the context of recurrence prediction, the *BRAF*− group has an AUC of 0.827, high sensitivity (92.9%), but a compromised specificity (51.8%). The *BRAF*+ subset has balanced metrics with an AUC of 0.817, sensitivity of 70.6%, and specificity of 72.1%. In the context of Hashimoto’s thyroiditis, the HT- group reaches an AUC of 0.814, with good sensitivity (82.8%) and moderate specificity (60.8%). However, the HT+ group’s metrics are modest with an AUC of 0.75, sensitivity of 50.0%, and specificity of 58.3%. Our findings support the pivotal role of the MMP9/miR-145 ratio in prognosis prediction with balanced accuracy in terms of *BRAF* mutation and Hashimoto’s thyroiditis.

#### 3.1.5. Survival Analysis

**Figure 3:**
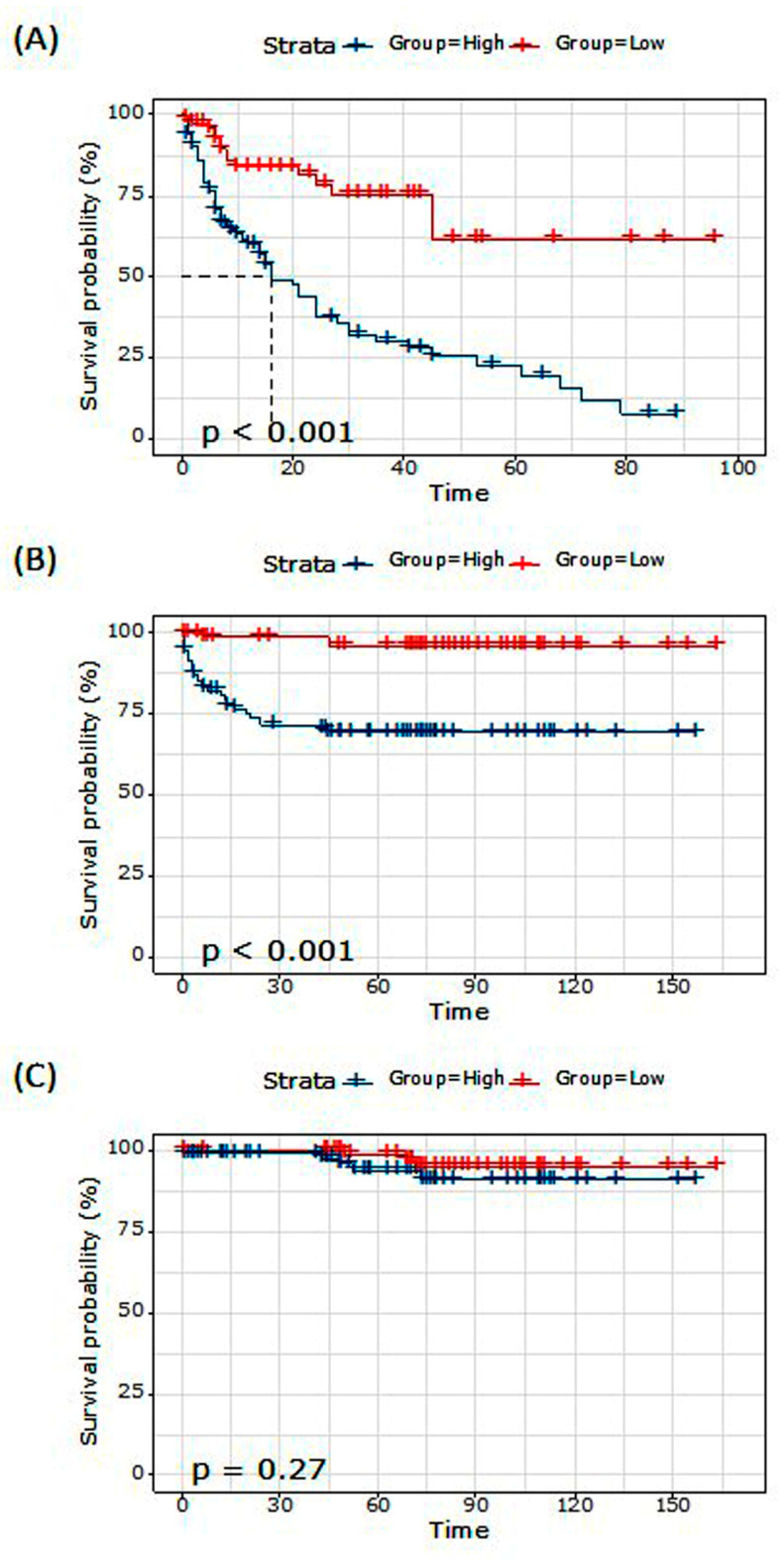
Kaplan–Meier curve for survival analysis. MMP9/miR-145 ratio was used to categorize patients at the cutoff of 15 into high expressors and low expressors. Survival times are presented in months on *x*-axis. Comparison was performed using log rank (Mantel–Cox) test. Significance was set at *p* < 0.05. (**A**) Relapse-free survival times. (**B**) Disease-free survival time. (**C**) Overall survival analysis.

Based on the best cutoff value of the MMP9/miR-145 ratio at 15, we categorized the patients into two groups. Kaplan–Meier survival curves were generated for three different clinical outcomes, namely relapse-free survival (RFS), disease-free survival (DFS), and overall survival (OS), as depicted in Figure 3. The overall mean relapse-free survival time was 44.20 months. Among patients with a high MMP9/miR-145 ratio, the mean survival time was 29.46 months (95% CI: 22.45–36.48), whereas for those with a low ratio, it was 68.47 months (95% CI: 55.97–80.97), *p* < 0.001 (Figure 3A). For DFS, the overall mean survival time was 135.82 months. The high-ratio group demonstrated a mean survival time of 112.17 months (95% CI: 98.27–126.08), while the low-ratio group presented a longer mean survival time of 158.58 months (95% CI: 152.54–164.62), *p* < 0.001 (Figure 3B). Regarding OS, the overall mean survival duration was 156.58 months. Patients in the high-ratio category showed a mean survival time of 147.40 months (95% CI: 139.20–155.59). Conversely, those in the low-ratio category had a mean survival time of 159.25 months (95% CI: 153.98–164.51), *p* = 0.27 (Figure 3C).

**Figure 4:**
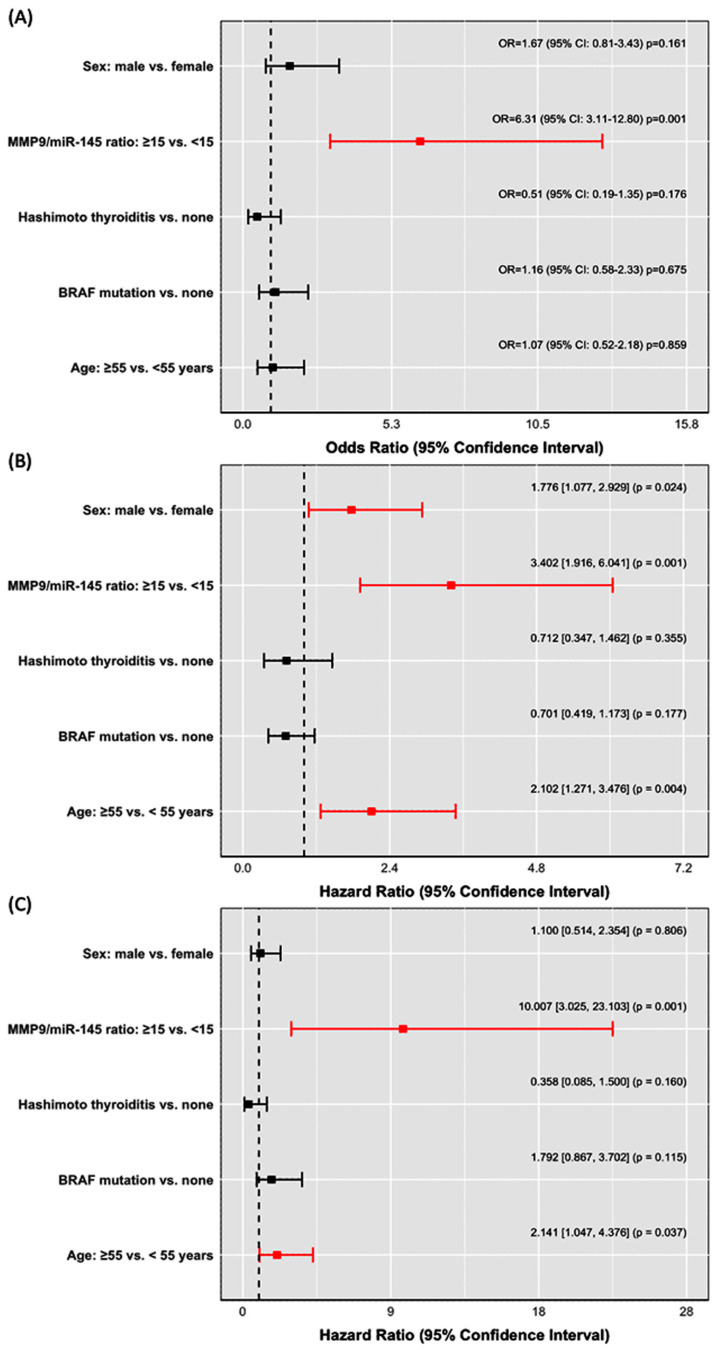
Impact of MMP9/miR-145 ratio in thyroid tissues on predicting disease outcomes at the time of surgery. (**A**) Risk factors predicting lymph node metastasis. Multivariate logistic regression analysis was employed, and odds ratio (OR) and 95% confidence intervals (CI) were reported. (**B**) Risk factors predicting disease progression and relapse. Cox regression model was performed, and hazards ratio (HR) and 95% CI were reported. (**C**) Risk factors predicting recurrence. Cox regression model was performed, and HR and 95% CI were reported.

Using binary logistic regression, a notable association was identified between the MMP9/miR-145 ratio of ≥15 and six-fold increased risk of LNM at presentation (OR = 6.31, 95% CI = 3.11–12.8, *p* = 0.001). Concurrently, multivariate Cox regression analyses indicated that the MMP9/miR-145 ratio is linked with a more than three-fold heightened risk of disease progression/relapse (HR = 3.40, 95% CI = 1.92–6.04, *p* = 0.001), which further escalates tenfold for recurrence risk (HR = 10.01, 95% CI = 3.03–23.10, *p* = 0.001) (Figure 4).

#### 3.1.6. Circulatory MMP9/miR-145 Ratio Expression Levels

**Figure 5:**
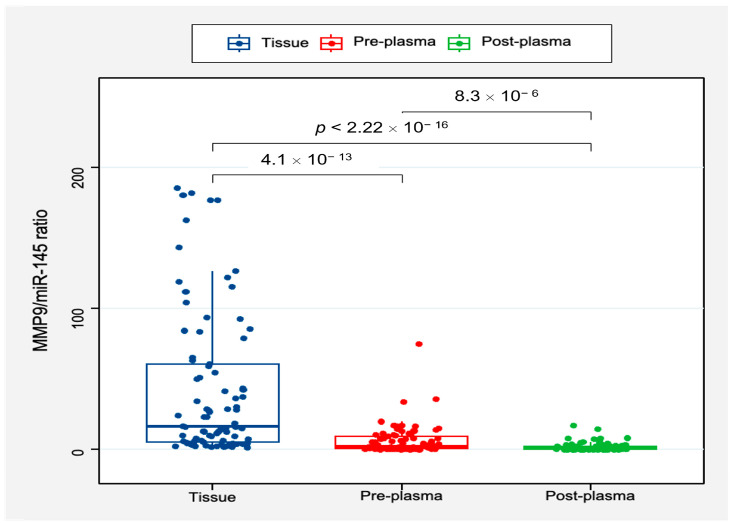
Circulatory expression of MMP9/miR-145 ratio in PTC patients. A comparative analysis of plasma and tissue samples of 86 PTC patients. Boxplot representing the median and interquartile range of the timepoint. Related-samples Friedman’s two-way analysis of variance by ranks test. Significance values have been adjusted by the Bonferroni correction for multiple tests.

In a cohort of 86 PTC patients with matched perioperative tissue and blood samples, there was a distinct difference between tissue and plasma levels of the MMP9/miR-145 ratio. The median MMP9/miR-145 ratio in resected thyroid tissues was markedly higher at 17.6 (IQR: 5.3–64.0) compared to the corresponding pre-operative plasma values, which presented a median level of 2.39 (IQR: 0.61–9.71), which might imply a higher local production or accumulation within the tumor microenvironment. Following surgical intervention, plasma MMP9/miR-145 ratio declined significantly to a median of 0.74 (IQR: 0.24–2.25), highlighting the diagnostic role of the ratio score as a biomarker for cancer (Figure 5). An observed strong correlation existed between preoperative plasma and thyroid tissue levels of the MMP9/miR-145 ratio (r = 0.808, *p* < 0.001). However, no correlation was found between the pre- and postoperative plasma levels (r = 0.076, *p* = 0.82).

#### 3.1.7. Clinical Implication of Preoperative MMP9/miR-145 Ratio in PTC Patients

Table 5 presents a breakdown of clinical associations to demonstrate variance in the MMP9/miR-145 ratio across diverse patient conditions. In the assessment of 86 PTC patients with corresponding perioperative tissue and blood samples, the expression levels of MMP9/miR-145 ratio in tissue were notably different from plasma levels (*p* < 0.05). Furthermore, post-operative assessments, conducted 3–5 days following tumor excision, showed that for certain subsets, such as those without lymph node metastasis (LNM) or those presenting with extrathyroidal extension (ETE), the difference between pre- and post-operative plasma levels was not statistically significant. Similarly, in patients who experienced recurrence, no significant variation in the ratio was observed post-operatively. ₓ 10 ^−13^ ₓ 10 ^−6^ 8 × 10^−6^.

**Figure 6:**
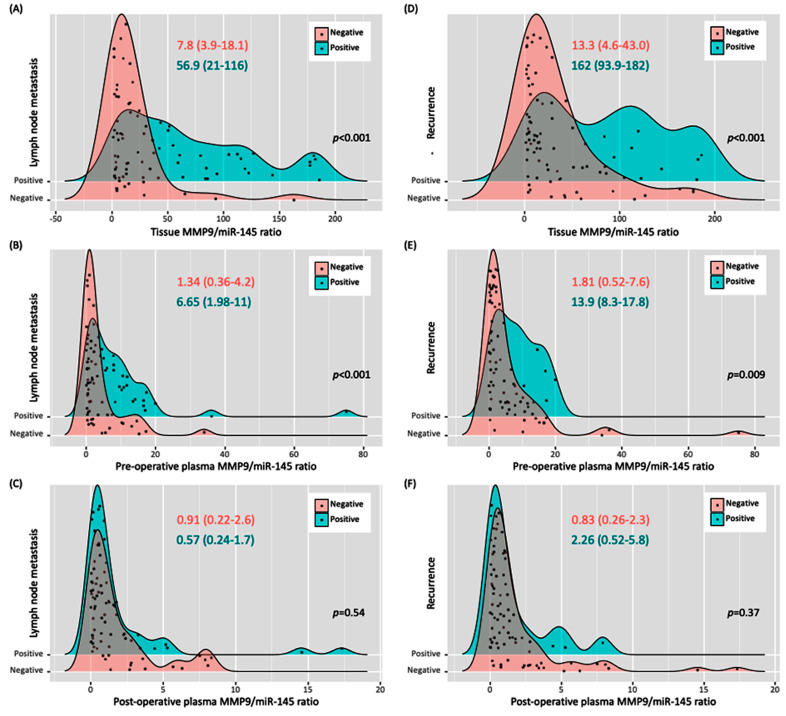
Comparative densitogram of MMP9/miR-145 ratios in PTC patients. Comparative analysis of patients based on the presence or absence of LNM and recurrence among 86 participants with PTC. Panels A to C differentiate between 42 patients with LNM and 44 patients without LNM. (**A**) Ratio in tissue. (**B**) Ratio in pre-operative plasma. (**C**) Ratio in immediate post-operative plasma. Meanwhile, panels D to F compare 17 patients with recurrence to the 69 without recurrence. (**D**) Ratio in tissue. (**E**) Ratio in pre-operative plasma. (**F**) Ratio in immediate post-operative plasma. Statistical analysis was conducted using the independent-samples Mann–Whitney U Test. A *p*-value of less than 0.05 was considered statistically significant.

As depicted in Table 5 and Figure 6, the pre-operative plasma levels of the MMP9/miR-145 ratio appear to be a crucial marker for assessing clinical outcomes in patients with PTC before surgery. Almost half (48.8%) of the patients presented with lymph node metastasis (LNM). Those with LNM had a considerably higher pre-operative plasma ratio (median: 6.65, IQR: 1.98–11.6) than the LNM-negative group (median: 1.34, IQR: 0.36–4.2). Furthermore, 22.1% of the studied cohort was identified with extrathyroidal extension (ETE). Those diagnosed with ETE exhibited a strikingly high pre-operative plasma ratio with a median value of 16.3 (IQR: 4.53–65.5). In contrast, individuals without ETE had a substantially lower ratio, with a median of 0.74 (IQR: 0.22–1.78). Another notable observation was related to disease progression or relapse, with 45.3% of the patients experiencing either condition. For this subgroup, the pre-operative plasma ratio was remarkably elevated, with a median value of 59.2 (IQR: 27.0–119). Patients who experienced recurrence (19.8%) demonstrated a higher pre-surgery plasma ratio (median: 13.9, IQR: 8.27–17.8) compared to non-recurrent cases, highlighting the potential prognostic value of the MMP9/miR-145 ratio. The small group of patients who died (4.7%) had a median pre-surgery plasma ratio of 6.5 (IQR: 5.8–14.1). However, this observation requires cautious interpretation given the limited number of cases.

#### 3.1.8. Over a 12–18-Month Post-Operative Duration, a Longitudinal Assessment was Conducted on the Initial Cohort of 86 PTC Patients

**Figure 7:**
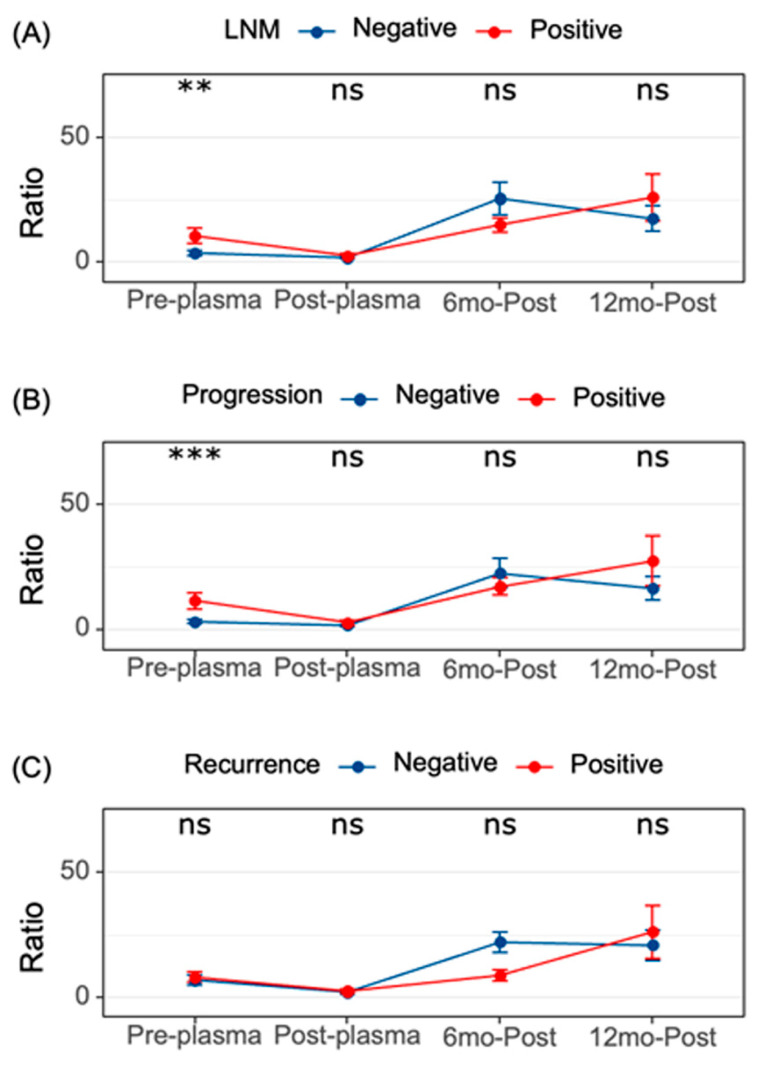
Evaluating the predictive capacity of plasma MMP9/miR-145 ratio. Subgroup analysis by (**A**) LNM: lymph node metastasis, (**B**) progression, and (**C**) recurrence to compare post-operative MMP9/miR-145 ratio values in circulation. The analysis was performed using the related-samples Friedman’s two-way analysis of variance by ranks test. All significant values were adjusted by the Bonferroni correction for multiple tests. ** *p* < 0.01, *** *p* < 0.001. ns: non-significant.

Over a 12–18-month post-operative duration, a longitudinal assessment was conducted on the initial cohort of 86 PTC patients. Within the initial 6 months following surgery, samples were collected from 69 patients, yielding a median MMP9/miR-145 ratio of 4.94 (IQR: 0.31–14.6). By the 12–18-month interval, 48 patients remained under study, reporting a median plasma ratio of 7.82 (IQR: 2.74–22.7). A thorough comparison was undertaken on these 48 patients, possessing complete data measurements. The results demonstrate marked disparities, with both the 6-month (*p* < 0.001) and 12–18-month figures showing notable deviation from immediate post-operative values, surpassing even pre-operative levels. However, the span between the 6-month and the 12–18-month readings revealed no discrete difference (*p* > 0.05). Figure 7 delineates how the ratio evolved over time, splitting data by nodal infiltration, progression/relapse, and recurrence. The data reveal a conspicuously higher ratio pre-operatively in patients manifesting nodal metastasis and those who later exhibited recurrence highlighting the predictive role of MMP9/miR-145 pre-operatively. Yet, in post-operative periods, they did not discernibly differentiate between patients with favorable versus unfavorable outcomes, thereby suggesting its limited applicability as a consistent biomarker for post-resection monitoring.

#### 3.1.9. Distinctive MMP9 Expression Patterns across Thyroid Tissue Types

**Figure 8:**
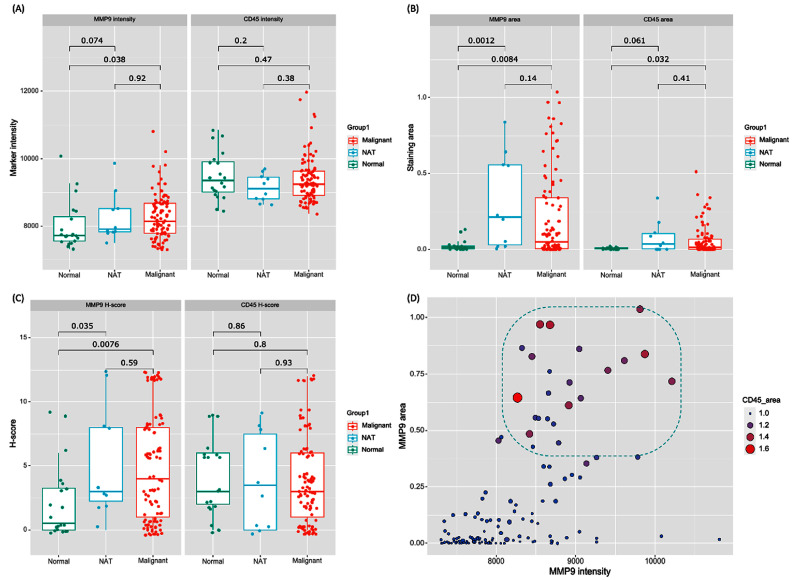
MMP9 expression patterns and correlation with immune infiltration across thyroid tissue types. (**A**) Boxplot showing MMP9 and CD45 expression intensity across normal, non-cancer adjacent tissues (NAT), and malignant thyroid tissues. (**B**) Boxplot showing the MMP9 and CD45 positive area across tissue types. Y axes represent the percentage area of the marker, divided by the nuclear area stained with DAPI area. (**C**) Boxplot showing H-scores of MMP9 and CD45 across the tissue types. The Kruskal–Wallis test was used for analysis followed by Mann–Whitney U test for pairwise comparisons. (**D**) Scatterplot showing the correlation between MMP9 area and MMP9 intensity for overall samples. Spearman’s correlation area was performed. Color gradient is based on the degree of immune cell infiltration as evidenced by CD45 percent area. NAT: non-cancer adjacent tissue.

An immunofluorescence analysis of tissue microarrays revealed distinctive MMP9 expression patterns across normal thyroid, adjacent non-tumor, and malignant tissues. A quantitative image analysis uncovered notable distinctions in MMP9 intensity and spatial distribution. The data show a statistically significant upregulation in MMP9 intensity in malignant tissues compared to normal thyroid (*p* = 0.038), suggesting overexpression during malignant transformation. However, MMP9 intensity in the non-cancer adjacent tissue (NAT) closely resembled that of malignant tissues (*p* = 0.92), with no significant difference from normal tissues (*p* = 0.07) (Figure 8A). Spatial mapping revealed a 10-fold higher median MMP9-positive area in malignant cores (0.096) versus normal tissues (0.01, *p* = 0.008), indicating localized overexpression in tumors. Interestingly, NAT areas resembled tumors spatially (median: 0.12, *p* = 0.14). The homogeneous MMP9 expression across tissue types implies limited utility as a standalone marker in delineating NAT from malignant thyroid (Figure 8B).

#### 3.1.10. Unraveling the Intricate Interplay between MMP9 Expression and Immune Infiltration

Analysis of CD45, an indicator of immune cell infiltration, showed a statistical difference between malignancy and normal samples (0.013 vs. 0.006, *p* = 0.032). However, there were no significant differences between malignant and NAT (*p* = 0.41) and NAT and normal tissues (*p* = 0.06) (Figure 8B). Based on proportion of stained cells and intensity, H-scores were estimated for both MMP9 and CD45. There was a significant increase in MMP9 expression in malignant tissues compared to normal tissues (*p* = 0.007). NAT also showed increased MMP9 expression compared to normal tissues (*p* = 0.035). However, the difference between malignant and NAT samples was not significant (*p* = 0.59). This pattern of MMP9 enrichment between tissue zones alludes to potential paracrine effects or field cancerization phenomena, wherein changes extend beyond visibly malignant areas. In contrast, CD45 H-scores showed no statistically significant differences between any of the tissue types: malignant vs. normal (*p* = 0.80); NAT vs. normal (*p* = 0.86); malignant vs. NAT (*p* = 0.93) (Figure 8C). This consistency in CD45 expression suggests the degree of immune involvement remains largely comparable across the examined thyroid tissue classes. Therefore, unlike MMP9 overexpression, immune activity may not be a primary distinguishing factor in thyroid cancer pathogenesis.

Further analysis revealed a significant positive correlation between MMP9 area and intensity (r = 0.561, *p* < 0.001). Cases with extensive MMP9 overactivity in large areas displayed markedly higher CD45+ immune cell densities, up to four-fold higher. The strong direct correlation between MMP9 and CD45 areas (r = 0.893, *p* < 0.001) indicates an inflammatory pro-tumoral microenvironment, where increased MMP9 expression might be fostering an environment that promotes tumor growth (Figure 8D). In summary, while MMP9 shows nuanced differential expression, CD45 immune infiltration appears largely consistent across tissue types. However, MMP9 overexpression correlates with increased inflammation, suggesting interconnected roles.

### 3.2. In Silico Data Analysis

#### 3.2.1. Genomic Structure of MMP9

MMP9, a type of gelatinase, is made up of five distinct domains: a signal peptide, a prodomain, a catalytic domain, a hinge region, and a hemopexin-like domain. The signal peptide is essential for directing the secretion of the MMP9 protein outside of cells. The prodomain, which includes a cysteine switch motif (PRCGXPD), binds to zinc ions, thereby hindering the binding of water molecules and keeping MMP9 in its inactive form. The regulation of MMP9 activation is critically dependent on this cysteine switch mechanism, allowing proteolytic activators like MMP3 and plasmin to activate MMP-9 via proteolysis or prodomain disruption. The catalytic domain is a crucial component that determines the proteolytic activity of MMP9. It is composed of the zinc ion binding domain and the active site, where specific amino acids contribute to enhancing the efficiency of the catalytic process. Additionally, three type-II fibronectin repeats facilitate the binding of MMP9 to large substrates such as elastin, collagen, and gelatin, a feature unique to gelatinases and not found in other MMPs. The hinge region of MMP-9 contains a unique O-glycosylated domain, also referred to as the collagen type V-like domain, due to its similarity to collagen. This domain is exclusive to the MMP-9 in the MMP-48 family and contributes to the flexibility of MMP-9, both within and between domains. Lastly, the hemopexin-like domain contributes to the specificity of MMP-9 among the MMP family and has several functions, including substrate interaction, inhibitor binding, cell surface receptor attachment, and facilitation of auto-activation.

#### 3.2.2. MMP9-miR-145 Interaction

**Figure 9:**
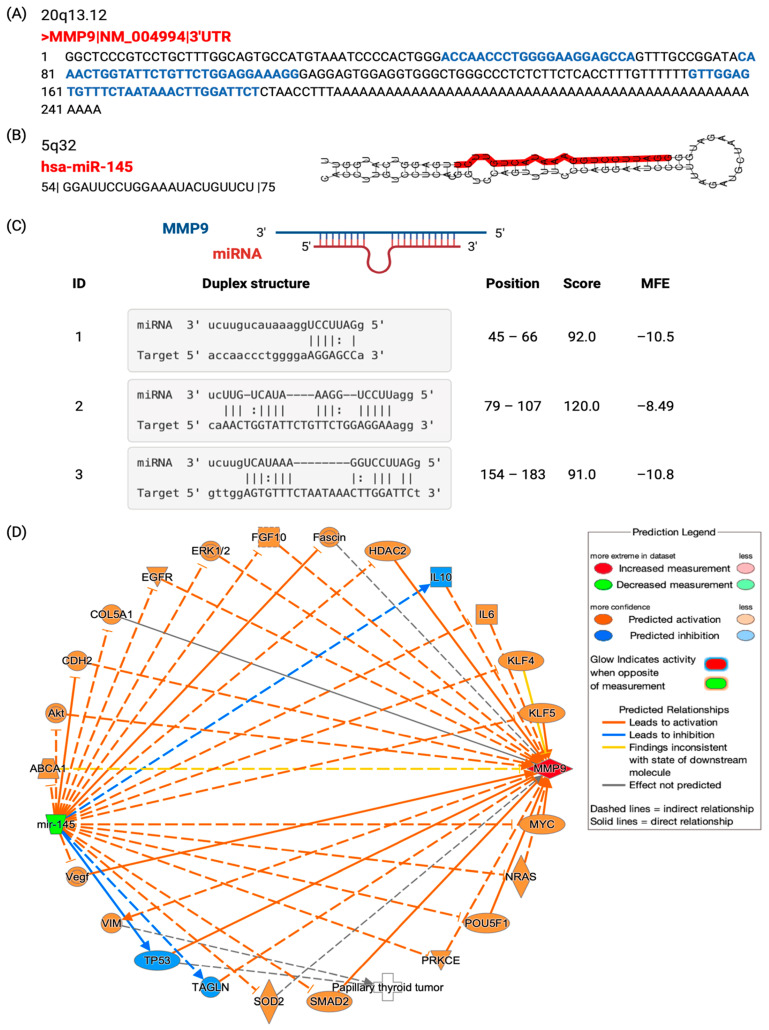
miRNA–gene interaction analysis. (**A**) MMP9 sequence at the 3′ untranslated region. (**B**) mature miRNA sequence. (**C**) Predicted regions of complement base pair between MMP9 and hsa-miR-145. Data source mirTarBase database. (**D**) Predicted functional effect of upregulation of MMP9 and downregulation of miR-145 in humans. Data source: Ingenuity Pathway Analysis version 01-22-01.

We identified three putative regions in MMP9 gene sequence that are complementary to the seed region of mature hsa-miR145 (Figure 9A–C). Transcriptomic analysis of some cancer and non-cancer datasets in GEO demonstrated negative correlation between the *MMP9* gene and miR-145. These included GSE19783 (breast cancer, *n* = 79 samples, r = −0.358, *p* < 0.001), GSE21032 (prostate cancer, *n* = 83, r = 0.332, *p* < 0.001), GSE19536 (breast cancer, *n* = 100, r = −0.297, *p* < 0.001), GSE28544 (breast cancer, *n* = 24, r = −0.42, *p* = 0.023), and GSE34608 (pulmonary tuberculosis and sarcoidosis, *n* = 20, r = −0.322, *p* = 0.08). Based on the IPA, from which we retrieved the connections from the literature, miR-145 can modulate MMP9 through various intermediate molecules. Figure 9D shows the prediction consequences of upregulation of MMP9 and downregulation of miR-145 in humans.

#### 3.2.3. The Link of MMP9 and miR-145 with Immunity in Thyroid Tissues: Insights from Multi-Dataset Analyses

**Figure 10:**
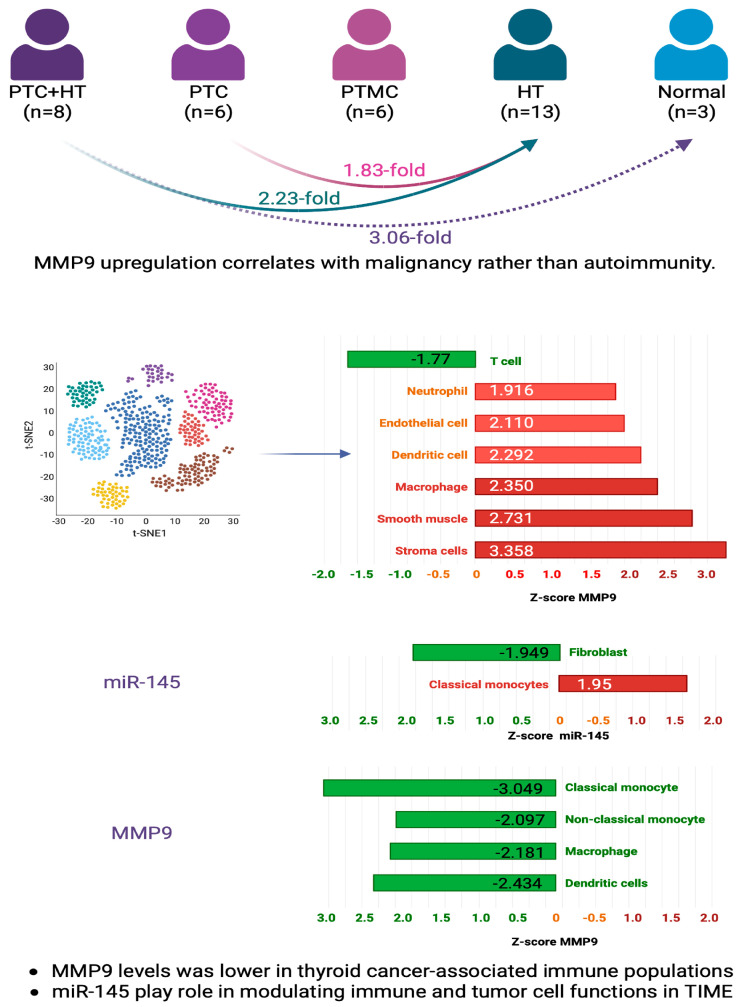
The quantitative relationships between MMP9 and miR-145 and immune cells in thyroid tissues (the data derived from the microarray dataset GSE138198 [29]). t-SNE: t-distributed stochastic neighbor embedding, an unsupervised non-linear dimensionality reduction technique for data exploration and visualizing high-dimensional data. The data presentation relate to single-cell RNA seq; each colored dot cluster represents the subtype of immune, stromal, and epithelial cell populations.

Several public datasets provide insight into the quantitative relationships between MMP9 and miR-145 and immune cells in thyroid tissues. In the microarray dataset GSE138198 (*n* = 36 samples) [29], including 13 Hashimoto’s thyroiditis (HT), 8 PTC with HT, 6 PTC without HT, 6 papillary microcarcinoma, and 3 normal thyroid samples, MMP9 expression was 1.83-fold higher in PTC without Hashimoto’s thyroiditis (HT) compared to HT alone (z = 1.83, *p* = 0.016). MMP9 levels were 2.23-fold higher in PTC with HT versus HT (z = 2.23, *p* = 0.006) and 3.06-fold higher in PTC with HT versus normal thyroid (z = 3.06, *p* = 0.002). This indicates MMP9 upregulation correlates with malignancy rather than autoimmunity (Figure 10).

In the single-cell RNA sequencing analysis GSE134355 (*n* = 2 samples), two samples of normal thyroid tissue were profiled [30]. Over 3000 individual cells were sequenced and grouped into nine major clusters corresponding to immune, stromal, and epithelial cell populations. Differential expression analysis was performed to compare MMP9 levels between each cluster and the combined remaining cells. MMP9 was enriched in thyroid-resident macrophage (z = 2.35, *p* < 0.001), neutrophil (z = 1.916, *p* = 0.007), stromal cell (z = 3.358, *p* = 0.004), conventional dendritic cell (z = 2.292, *p* = 0.012), smooth muscle cell (z = 2.731, *p* = 0.001), and endothelial cell clusters (z = 2.11, *p* = 0.51) compared to other cell clusters, highlighting cell type-specific regulation. However, MMP9 was downregulated in T cell clusters (z = −1.77, *p* = 0.17).

In the pan-cancer single-cell RNA sequencing dataset GSE154763 investigating tumor-infiltrating myeloid cells (TIMs) across multiple cancer types, including thyroid cancer [31], scRNA-seq data weregenerated from thyroid tissue samples of 10 thyroid cancer patients. Over 5000 individual TIMs were profiled and grouped into clusters corresponding to subpopulations like macrophages, mast cells, and monocytes. MMP9 levels were lower in thyroid cancer-associated immune subpopulations like conventional dendritic cells (z = −2.434, *p* < 0.001), mast cells (z = −1.879, *p* < 0.001), classical monocytes (z = −3.049, *p* < 0.001), non-classical monocytes (z = −2.097, *p* < 0.001), and macrophages (z = −2.181, *p* < 0.001) versus respective levels in other clusters. This contrasts with the overexpression seen in normal thyroid immune cells. Within the classical monocyte cluster, miR-145 expression was higher compared to other cell types (z = 1.95, *p* = 0.016). miR-145 levels were lower in the fibroblast cluster versus other clusters (z = −1.949, *p* = 0.10). These findings suggest miR-145 may play a role in modulating immune and tumor cell functions in the thyroid cancer microenvironment. Together, these results demonstrate complex context-dependent associations between MMP9 and tumor-infiltrating immune cells in thyroid cancer. Further research is required to elucidate the functional immune interplay regulating MMP9 expression.

### 3.3. Systematic Review

#### 3.3.1. Functional Analysis of MMP9 and miR-145

Although MMP9 is known to be involved in thyroid cancer pathogenesis, the specific molecular mechanisms remain to be elucidated. Some potential mechanisms that have been proposed include degradation of extracellular matrix proteins, promotion of angiogenesis, cleavage of growth factors and receptors, and epigenetic changes. As an endopeptidase, MMP9 can degrade type IV collagen and denatured collagen, which are major components of the basement membrane surrounding tumor cells. Breakdown of the basement membrane is thought to facilitate tumor invasion and metastasis [32,33]. MMP9 enables the release and activation of pro-angiogenic factors like vascular endothelial growth factor (VEGF). MMP9 and VEGF act synergistically to stimulate new blood vessel formation (angiogenesis), which supports tumor growth and progression [34,35]. MMP9 is able to proteolytically process many growth factor precursors, growth factor binding proteins, cell surface receptors like receptor tyrosine kinases, and cell adhesion molecules. This can alter signaling pathways in ways that promote uncontrolled growth and metastasis [36]. In addition, some studies have found decreased methylation of the *MMP9* gene promoter in thyroid cancer, which could lead to increased *MMP9* transcription [37].

In terms of specific signaling pathways implicated in thyroid cancer, MMP9 has been shown to be an important mediator in many pathways that regulate invasion and metastasis. One such pathway involves the TR4 nuclear receptor, circular RNA FNLA, and miR-149-5p microRNA. The circular RNA FNLA acts as a sponge for miR-149-5p, a microRNA that suppresses MMP9, thereby indirectly increasing MMP9 levels and promoting tumor invasion and metastasis [38]. Another pathway involves the SOX12 transcription factor, which activates the expression of *POU2F1* and *POU3F1*, subsequently increasing the expression of *MMP9* and promoting the invasion and metastasis of PTC cells [39]. Similarly, the long non-coding RNA DUXAP10 upregulates the Akt/mTOR signaling pathway, leading to increased *MMP9* expression and promoting tumor progression and metastasis [40]. In another signaling pathway, the lysine-specific demethylase KDM1A decreases the expression of *TIMP-1*, an endogenous inhibitor of MMP9, leading to increased MMP9 activity and promoting tumor invasion [41]. The *ALOX5* gene, encoding the 5-lipoxygenase enzyme involved in the metabolism of arachidonic acid, also plays a role. The overexpression of *ALOX5* increases *MMP9* expression and activity, contributing to PTC progression [42]. The Rho-associated protein kinase 1 (ROCK1) is another regulator of cell shape, motility, and tumor invasion. Increased *ROCK1* expression enhances *MMP9* expression and activity, promoting PTC invasion and metastasis [43]. Furthermore, the calcium-binding protein S100A4 is involved in tumor progression and metastasis. Overexpression of S100A4 increases *MMP9* expression and activity, promoting PTC invasion [44]. The protein Enigma activates the PI3K/AKT signaling pathway, increasing *MMP9* expression and promoting PTC progression and metastasis [45]. Lastly, the Interleukin-17 receptor B (IL-17RB) activates the ERK1/2 signaling pathway, increasing *MMP9* expression and promoting PTC invasion and metastasis [46]. Elucidating these pathways may reveal promising therapeutic targets for suppressing MMP9-mediated tumor progression in thyroid cancer.

In thyroid cancer, miR-145 has been identified as a downregulated tumor suppressor microRNA. Functional and preclinical studies have shown that AKT3, ZEB1, ZEB2, DUSP6, and RAB5C are direct targets of miR-145 [10,11,12,47,48]. These targeted proteins and genes play critical roles in cell proliferation, apoptosis, motility, angiogenesis, invasion, and metastasis in thyroid cancer. For example, AKT is vital in the PI3K/Akt signaling pathway [49], ZEB1 modulates the PD-1/PD-L1 checkpoint and cancer stem cell maintenance [50,51,52], ZEB2 triggers epithelial-mesenchymal transition and activates the Wnt/beta-catenin pathway [53,54], RAB5C regulates angiogenesis and cell migration [55,56,57], and DUSP6 is involved in thyroid tumorigenesis by regulating the ERK1/2 pathway [58]. Three ncRNAs—TUG1, n384546, and circNUP214—can sponge and suppress miR-145, leading to cancer progression and metastasis [10,11,59]. Anti-miR-145 can partially reverse the effects of lncRNA knockdown by regulating AKT3 expression. Furthermore, miR-145 modulates the NF-κB pathway in PTC cells [60]. miR-145 polymorphisms are associated with higher thyroid cancer risk. Functional enrichment analysis using Diana tools revealed that miR-145-5p negatively regulates multiple genes in the thyroid cancer signaling pathway [hsa05216], including neuroblastoma RAS proto-oncogenes (NRAS), MYC BHLH transcription factor (MYC), tropomyosin 3 (TPM3), catenin beta 1 (CTNNB1), and cyclin D1 (CCND1). Therefore, miR-145 loss promotes thyroid cancer aggression by de-repressing crucial mediators of proliferation, EMT, invasion, metastasis, and angiogenesis.

#### 3.3.2. MMP9 and miR-145 Expression in Thyroid Cancer

**Figure 11:**
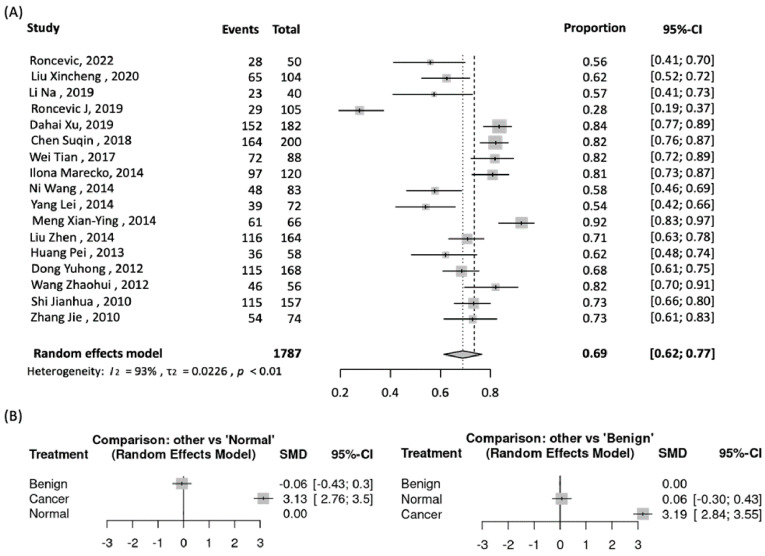
Meta-analysis on the tissue and circulatory MMP9 levels in thyroid tumors. (**A**) Pooled analysis of MMP9 expression level in tissues of PTC patients. (**B**) Network meta-analysis comparing the circulatory level of MMP9 in patients with malignant and benign thyroid diseases and healthy controls [21,61,62,63,64,65,66,67,68,69,70,71,72,73,74,75,76].

Published studies demonstrated elevated MMP9 levels in thyroid cancer tissues, cells, and body fluids compared to benign diseases or normal controls. Studies have analyzed MMP9 protein and mRNA levels in thyroid tissues using immunohistochemistry, enzyme-linked immunosorbent assay (ELISA), gelatin zymography, Western blotting, and polymerase chain reaction (PCR). Most report significantly higher MMP9 expression in thyroid cancer specimens than in normal tissues or benign nodules [21,61,62,63,64,65,66,67,68,69,70,71]. Our meta-analysis showed that high MMP9 level is prevalent in 69% (95% CI: 62–77%) of PTC tissue specimens (Figure 11A).

In addition to tissue samples, several studies analyzed MMP9 levels in body fluids of thyroid cancer patients [65,72,73,74,75,76]. Multiple studies have reported significantly higher serum/plasma MMP9 levels in PTC patients than those with benign thyroid diseases and healthy controls. Our network meta-analysis of six studies (418 PTC patients, 221 benign diseases, 121 controls) using frequentist methods found that patients with PTC had significantly increased serum/plasma MMP9 versus both benign disease (SMD: 3.19, 95% CI: 2.84–3.55) and healthy controls (SMD: 3.13, 95% CI: 2.76–3.50) (Figure 11B). However, serum MMP9 levels were not different between benign and malignant pathology in two studies [73,76], highlighting the need to account for confounders. Data on circulating miR-145 levels in TC patients is limited, representing an area requiring further investigation.

In vitro studies have investigated intracellular MMP9 levels in thyroid cancer cell lines. Using Western blotting and RT-PCR, Li et al. discovered increased MMP9 protein levels in PTC (IHH-4: 6.59 ± 1.24), follicular thyroid cancer (FTC) (FTC-133: 5.10 ± 0.91), and anaplastic thyroid cancer (ATC) (8505C: 5.42 ± 0.86) cell lines compared to normal thyroid follicular epithelial (HT-ori3: 1.01 ± 0.43) cells. Additionally, the RT-PCR revealed that the MMP9 mRNA levels were also elevated in the cancer cell lines: IHH-4 cells had an mRNA level of 4.56 ± 0.61, FTC-133 cells had 3.41 ± 0.42, and 8505C cells had 2.79 ± 0.26, compared to the HT-ori3 cells, which had an mRNA level of 1.01 ± 0.09 [22]. This pattern of elevated MMP9 expression in thyroid cancer cells aligns with previous observations of pronounced MMP9 upregulation in actual patient tumors and highlights the importance of further exploring MMP9 as a potential target for thyroid cancer therapy.

#### 3.3.3. Associations with Clinicopathological Features

Several studies have examined the associations between MMP9 expression levels and clinicopathological parameters reflective of TC progression and prognosis (Table 6) [23,61,62,63,64,73,77,78]. Higher MMP9 expression has been consistently associated with the presence of lymph node metastasis compared to PTC without metastasis in multiple studies [21,22,62,65,67,79,80]. Dragutinovic et al. also found increased MMP9 immunostaining in the more poorly differentiated classical PTC subtype versus the follicular variant [81]. Higher MMP9 levels have additionally been linked to advanced TNM stage in PTC [62,65,67,80], extrathyroidal extension [21,61,64,65,75,79], and larger tumor size [62,65,67] in PTC. Clinically, MMP9 activity distinguishes between sporadic and radiation-induced PTC, with greater MMP9 expression in post-Chernobyl tumors indicating more aggressive disease [82]. Circulating MMP9 levels have also positively correlated with parameters like tumor size, extrathyroidal extension, lymph node metastasis, advanced TNM stage, distant metastasis, and lymphovascular invasion in PTC patients [65,74,75]. A meta-analysis of 2561 PTC patients confirmed MMP9 overexpression was significantly associated with larger tumor size in PTC (OR: 1.69, 95% CI: 1.13–2.52), higher TNM stage in PTC (OR: 2.95, 95% CI: 2.10–4.13), and lymph node metastasis in PTC (OR: 3.92, 95% CI: 2.71–5.65) [62]. For example, Liu et al. found that patients with central/lateral cervical lymph node metastasis had higher MMP9 levels and more advanced TNM stages [77]. Moreover, MMP9 activation, rather than just expression, may better predict metastatic potential [21]. Regarding miR-145, our prior work showed it is downregulated in PTC, and lower miR-145 expression is associated with more aggressive clinicopathological features, poorer survival, increased recurrence, and worse prognosis [9]. Thus, miR-145 may be a useful prognostic biomarker for high-risk thyroid cancer.

#### 3.3.4. Longitudinal Studies for Prognostication

Several longitudinal studies evaluated changes in MMP9 levels before and after surgery or during follow-up of TC patients. These studies provide evidence that MMP9 may have utility as a prognostic biomarker to predict outcomes [65,72,73,74,77,79]. Multiple studies found higher preoperative MMP9 levels in thyroid cancer than post-surgery, indicating surgical resection leads to a decrease in MMP9. For example, Zhang et al. reported serum MMP9 levels were significantly higher in TC before surgery (134.70 ± 32.52 ng/mL) compared to 1 month after surgery (51.46 ± 18.34 ng/mL) [74]. With longer follow-up, Pan et al. observed higher MMP9 levels preoperatively (299.98 ± 70.48 ng/mL), which gradually declined over 12 months post-surgery to 169.07 ± 64.16 ng/mL [72]. Notably, some studies linked elevated MMP9 with a worse prognosis. Xu et al. found higher tissue and serum MMP9 expression in patients with structurally persistent or recurrent disease during follow-up than in disease-free individuals [65,73]. Similarly, Buergy et al. noted increasing MMP9 levels accompanied by metastasis and recurrence [79]. In summary, longitudinal studies demonstrate that MMP9 levels decrease after TC treatment and surgical resection. MMP9 levels may increase with tumor persistence, recurrence, or progression during follow-up. Although more extensive studies are warranted, existing evidence suggests MMP9 has potential as a prognostic biomarker to identify high-risk TC patients needing closer surveillance or aggressive therapy.

While several cross-sectional studies have reported that miR-145 is downregulated in TC tissues and blood samples compared to controls [9], there is a gap in knowledge regarding how miR-145 expression changes perioperatively or during long-term follow-up of thyroid cancer patients. To our knowledge, there has been no exploration of how miR-145 levels change before and after surgical resection for thyroid cancer or how they fluctuate relative to persistent/recurrent disease or during treatment and surveillance. The lack of longitudinal data limits our understanding of miR-145 utility as a prognostic marker for aggressive disease or predictor of recurrence. It remains unclear whether declining miR-145 levels over time could signal the emergence of minimal residual disease or identify patients at high risk of relapse needing closer monitoring. Notably, our study is the first to evaluate dynamic changes in miR-145 expression perioperatively and during postoperative follow-up of TC patients. We analyzed the fluctuations of miR-145 and MMP9 in blood and tissues before surgery, in the immediate postoperative period, and over long-term surveillance relative to patient outcomes, as discussed previously in Section 3.1.8.

#### 3.3.5. Impact of MMP9 Inhibition and miR-145 Activation on Thyroid Cancer

Current standard treatments for thyroid cancer include surgery, radioactive iodine therapy, TSH suppression, and chemotherapy. These limitations include overtreatment, side effects, and poor efficacy in advanced disease. MMP9 inhibitors and/or miR-145 activators represent a potential supplemental therapy approach. MMP9 is typically overexpressed in thyroid cancer and promotes processes like invasion and metastasis. Its inhibition could target signaling pathways involved in tumor progression. Simultaneously, miR-145, a tumor suppressor miRNA, has shown anti-tumor effects in various cancers, indicating that its activation may enhance responses to existing therapies and reverse therapeutic resistance. Both natural and synthetic MMP9 inhibitors have been investigated in thyroid cancer models. Plant-derived natural inhibitors such as curcumin, quercetin, and ginsenosides have demonstrated the ability to decrease MMP9 expression, as well as viability, migration, invasion, metastasis, and epithelial-mesenchymal transition (EMT) in thyroid cancer cells. Non-natural inhibitors, including valproic acid, sevoflurane, and celecoxib, have also been shown to reduce MMP9 levels and inhibit thyroid cancer progression. Additionally, other therapeutic approaches, such as non-thermal plasma, have been found to inhibit MMP9 activity and metastasis [23].

Curcumin, a polyphenol from turmeric, reduced the viability, migration, and epithelial-mesenchymal transition (EMT) of K1 PTC cells by downregulating MMP9 expression and activity. The underlying mechanisms include the upregulation of E-cadherin and the downregulation of N-cadherin and vimentin [64,65]. It downregulates hypoxia-induced MMP9 via HIF-1 to reduce migration and upregulates miR-301a-3p to directly target STAT3 and reduce MMP9. Quercetin, a common flavonoid found in fruits and vegetables, inhibited MMP9 activity and TC cell migration, invasion, and EMT. It also increases the expression of the sodium iodide symporter, enhancing radio-iodine uptake. These effects were mediated by modulating the MAPK, PI3K/AKT, and Wnt/β-catenin signaling pathways [85]. Ginsenoside Rg3, a component of ginseng, was shown to suppress MMP9 expression and TC metastasis by modifying actin cytoskeletal organization and integrin signaling [86]. Another compound, the pentacyclic triterpene taraxasterol, inhibited MMP9 expression, thereby reducing PTC cell migration, invasion, and EMT. These effects were mediated by inhibiting TGF-β1-induced epithelial-mesenchymal transition [87]. Valproic acid, an antiepileptic drug and histone deacetylase inhibitor, reduced MMP2/MMP9 levels in anaplastic thyroid cancer cells, promoting redifferentiation [88]. Sevoflurane, an anesthetic agent, inhibited miR-155, thereby reducing MMP2/MMP9 expression and, subsequently, migration and invasion in PTC cells [89]. Celecoxib, an anti-inflammatory COX-2 inhibitor, reduced MMP9, VEGF, and growth in medullary thyroid cancer cells and mouse models [90].

The synthetic MMP9/2 inhibitor SB-3CT reduced melanoma and lung cancer growth and metastasis in xenograft models by decreasing MMP9 expression [91,92]. The selective MMP9 activation inhibitor JNJ0966 suppressed melanoma metastasis by allosterically inhibiting MMP9 zymogen activation [93]. The dual MMP9/12 inhibitor AZD1236 inhibited lung and colon cancer metastasis in xenografts through coordinated inhibition of both MMPs [94]. Non-thermal plasma decreased MMP2/MMP9 activity and cytoskeletal changes, inhibiting invasion and metastasis in PTC cells [95]. Inhibition of MMP9 suppresses processes such as proliferation, EMT, migration, invasion, angiogenesis, and others that drive thyroid tumor growth and spread. It can also promote apoptosis and redifferentiation in cancer cells. By inhibiting these tumor-promoting effects, MMP9 inhibitors demonstrate potential as targeted adjunctive therapies for thyroid cancer. Both natural and synthetic agents have shown preclinical efficacy in suppressing disease progression through multiple mechanisms. More research is needed to evaluate MMP inhibitors as potential supplemental targeted therapies, especially for advanced thyroid cancers.

miR-145 acts as a tumor suppressor miRNA, and its replacement therapy showed anti-tumor effects in animal models of colon carcinoma, prostate cancer, etc. [9]. miR-145 can enhance the response to chemotherapeutic drugs like sorafenib, methotrexate, and docetaxel in the liver, osteosarcoma, and prostate cancer [96,97,98]. It reversed therapeutic resistance through different mechanisms in various cancers. In lung cancer, it increased sensitivity to gefitinib by targeting ADAM19 [99]. In esophageal cancer, it sensitized cells to cisplatin by inhibiting the PI3K/AKT pathway [100]. In colorectal cancer, it antagonized SNAI1-mediated stemness and radiation resistance [101] and also reversed 5-FU resistance by targeting RAD18 [102]. In glioblastoma, it enhanced chemosensitivity to desmethoxycurcumin [103]. Therefore, miR-145 may serve as a biomarker for drug resistance, and its therapeutic upregulation could improve response to conventional and targeted cancer therapies. More research is needed to fully evaluate the potential of miR-145 in reversing drug resistance in different cancers through diverse mechanisms. Its role as a sensitizer to therapy is promising and warrants further investigation.

In conclusion, the potential impacts of MMP9 inhibition and miR-145 activation on thyroid cancer are promising. MMP9 inhibitors, both natural and synthetic, have demonstrated the ability to inhibit various processes that drive thyroid tumor growth and spread, thus representing potential targeted adjunctive therapies for thyroid cancer. On the other hand, miR-145 has shown the capacity to enhance the response to chemotherapeutic drugs and reverse therapeutic resistance in various cancers, making it a promising biomarker for drug resistance and a potential sensitizer to therapy. Further research is necessary to comprehensively evaluate these potential supplemental targeted therapies, especially for advanced thyroid cancers.

## 4. Discussion

This study provides compelling evidence that the MMP9/miR-145 expression ratio holds promise as a versatile biomarker for thyroid cancer diagnosis and prognosis. We demonstrated consistent upregulation of MMP9 and reciprocal downregulation of miR-145 in malignant tissues compared to normal thyroid controls. The integrated MMP9/miR-145 ratio was significantly elevated in tumors, with a median 17.6-fold increase over non-tumor tissue. This indicates the combined ratio better delineates benign from malignant pathology than individual markers.

Critically, the circulating MMP9/miR-145 ratio was also elevated preoperatively in patients exhibiting aggressive disease characteristics, including lymph node metastasis, extrathyroidal extension, progression/relapse, and recurrence. The preoperative plasma ratio displayed high predictive accuracy for nodal metastasis (AUC = 0.791) and disease progression (AUC = 0.812). Patients with a high tissue ratio (>15) had a six-fold greater risk of nodal metastasis and over three-fold increased risk of relapse. This reveals the MMP9/miR-145 ratio’s utility for preoperative risk stratification to guide optimal treatment intensity.

Notably, the MMP9/miR-145 ratio maintained balanced accuracy across cohorts stratified by *BRAF* mutation status and Hashimoto’s thyroiditis. MMP9 and miR-145 levels individually can vary based on genetic and autoimmune backgrounds. However, the integrated ratio overcomes this heterogeneity, serving as a unified biomarker generalizable across diverse thyroid cancer subpopulations regardless of etiology. This represents a key advantage over individual markers.

Analysis of the ratio rendered better prediction value demonstrated in ROC analysis compared to each alone. The ratio showed good prediction for recurrence at the time of surgery in tissues and blood preoperatively. While there was a significantly higher ratio in cohorts who developed recurrence, normalization of the ratio following total thyroidectomy occurred for all cohorts and there was no significant difference between those who recurred and those who did not. Therefore, we recommend discovering ancillary markers for real-time postoperative surveillance, as the currently used thyroglobulin assay has limitations and low sensitivity [104].

Our longitudinal data reveal immediate declining MMP9/miR-145 levels after tumor resection. While this strongly predicts preoperative disease aggression, dynamic trends indicate decreasing clinical applicability for long-term post-thyroidectomy surveillance.

This pioneering study is the first to demonstrate the clinical merit of concurrently evaluating MMP9 and miR-145 as a unified expression ratio. This ratio captures the intricate molecular interplay between these two biomarkers better than in isolation. Future research can explore integration with other relevant markers like thyroglobulin to develop multi-parametric prognostic signatures.

Diving in related studies published previously with a supportive systematic analysis confirmed the essential roles of MMP9 and miR-145 play in thyroid cancer (Section 3.3.1 and Section 3.3.2), association of their expression with the clinicopathological features of thyroid cancer (Section 3.3.3), and their prognostication utility (Section 3.3.4).

In summary, this study establishes the MMP9/miR-145 ratio as a promising biomarker in thyroid cancer management that may guide preoperative decision making regarding the optimal extent of surgical resection and adjuvant therapies. Widespread validation is warranted to support the clinical translation of the MMP9/miR-145 ratio as a broadly applicable prognostic tool for the personalized management of heterogeneous thyroid cancer patients.

This study has some limitations. The cohort was relatively small, from a single institution, and limited to PTC histology. More extensive multi-center prospective studies encompassing diverse ethnicities and histologic subtypes are needed to validate findings. The longitudinal follow-up duration was restricted to 12–18 months, so more long-term outcome data are necessary to capture long-term recurrence events. Standardization of the detection methodology and accounting for technical/pre-analytical variables can enhance reproducibility for clinical application. Finally, analysis of circulating levels before and after radioactive iodine therapy may provide insights into treatment response prediction.

## 5. Conclusions

In conclusion, this study demonstrates that the MMP9/miR-145 ratio is a promising tissue and circulating biomarker that can enhance diagnostic accuracy, postoperative prognostication, and risk stratification in thyroid cancer patients. The ratio displayed efficacy in diverse patient groups regardless of *BRAF* mutation or autoimmune thyroiditis status, overcoming genetic and immunological heterogeneity. While the preoperative plasma ratio predicts high-risk disease features, its utility for long-term post-surgical monitoring and surveillance appears limited based on declining postoperative trends. Further large-scale validation across multi-ethnic cohorts is warranted to support clinical translation of the MMP9/miR-145 ratio. Widespread adoption can aid surgical planning and personalized therapy decisions for thyroid cancer patients. Overall, concurrent evaluation of MMP9 and miR-145 as an integrated biomarker ratio holds significant potential to improve prognostication and individualized management.

## Figures and Tables

**Table 1:** Baseline characteristics of thyroid cancer cohorts (*n* = 175) according to *BRAF* mutation and Hashimoto’s thyroiditis.

Patient Characteristics	Levels	*BRAF*^V600E^ Mutation		*p*-Value	Hashimoto’s Thyroiditis		*p*-Value
		Negative	Positive		Negative	Positive	
Total number		97	78		149	26	
Demographic data							
Age, years	<55 years	71 (73.2%)	48 (61.5%)	0.11	101 (67.8%)	18 (69.2%)	0.88
	≥55 years	26 (26.8%)	30 (38.5%)		48 (32.2%)	8 (30.8%)	
Sex	Female	60 (61.9%)	59 (75.6%)	0.07	101 (67.8%)	18 (69.2%)	0.88
	Male	37 (38.1%)	19 (24.4%)		48 (32.2%)	8 (30.8%)	
Obesity	Negative	6 (6.2%)	8 (10.3%)	0.40	12 (8.1%)	2 (7.7%)	0.95
	Positive	91 (93.8%)	70 (89.7%)		137 (91.9%)	24 (92.3%)	
Smoking	Negative	87 (89.7%)	73 (93.6%)	0.42	136 (91.3%)	24 (92.3%)	0.86
	Positive	10 (10.3%)	5 (6.4%)		13 (8.7%)	2 (7.7%)	
Hepatitis C virus	Negative	50 (51.5%)	49 (62.8%)	0.17	81 (54.4%)	18 (69.2%)	0.20
	Positive	47 (48.5%)	29 (37.2%)		68 (45.6%)	8 (30.8%)	
Genomic mutation							
RAS gene mutation	Negative	86 (88.7%)	74 (94.9%)	0.18	141 (94.6%)	19 (73.1%)	**0.002**
	Positive	11 (11.3%)	4 (5.1%)		8 (5.4%)	7 (26.9%)	
Pathological data							
Laterality	Unilateral	64 (0.7%)	56 (0.7%)	0.51	100 (0.7%)	20 (0.8%)	0.37
	Bilateral	33 (0.3%)	22 (0.3%)		49 (0.3%)	6 (0.2%)	
Focality	Unifocal	86 (0.9%)	59 (0.8%)	**0.027**	121 (0.8%)	24 (0.9%)	0.26
	Multifocal	11 (0.1%)	19 (0.2%)		28 (0.2%)	2 (0.1%)	
T stage	T1a	21 (21.6%)	26 (33.3%)	**<0.001**	39 (26.2%)	8 (30.8%)	0.75
	T1b	17 (17.5%)	29 (37.2%)		39 (26.2%)	7 (26.9%)	
	T2	54 (55.7%)	16 (20.5%)		60 (40.3%)	10 (38.5%)	
	T3	2 (2.1%)	6 (7.7%)		8 (5.4%)	0 (0%)	
	T4	3 (3.1%)	1 (1.3%)		3 (2%)	1 (3.8%)	
N stage	N0	54 (55.7%)	47 (60.3%)	0.64	83 (55.7%)	18 (69.2%)	0.28
	N1	43 (44.3%)	31 (39.7%)		66 (44.3%)	8 (30.8%)	
M stage	M0	87 (89.7%)	58 (74.4%)	**0.009**	121 (81.2%)	24 (92.3%)	0.26
	M1	10 (10.3%)	20 (25.6%)		28 (18.8%)	2 (7.7%)	
Pathology stage	Stage I	69 (71.1%)	46 (59%)	**0.027**	95 (63.8%)	20 (76.9%)	0.54
	Stage II	6 (6.2%)	8 (10.3%)		12 (8.1%)	2 (7.7%)	
	Stage III	10 (10.3%)	2 (2.6%)		11 (7.4%)	1 (3.8%)	
	Stage IVA	4 (4.1%)	7 (9%)		11 (7.4%)	0 (0%)	
	Stage IVB	2 (2.1%)	1 (1.3%)		2 (1.3%)	1 (3.8%)	
	Stage IVC	6 (6.2%)	14 (17.9%)		18 (12.1%)	2 (7.7%)	
Extrathyroidal extension	Negative	78 (80.4%)	59 (75.6%)	0.47	119 (79.9%)	18 (69.2%)	0.30
	Positive	19 (19.6%)	19 (24.4%)		30 (20.1%)	8 (30.8%)	
Perineural invasion	Negative	94 (96.9%)	77 (98.7%)	0.63	145 (97.3%)	26 (100%)	0.39
	Positive	3 (3.1%)	1 (1.3%)		4 (2.7%)	0 (0%)	
Extranodal extension	Negative	94 (96.9%)	69 (88.5%)	**0.036**	138 (92.6%)	25 (96.2%)	0.51
	Positive	3 (3.1%)	9 (11.5%)		11 (7.4%)	1 (3.8%)	
Lymphovascular invasion	Negative	86 (88.7%)	65 (83.3%)	0.38	127 (85.2%)	24 (92.3%)	0.54
	Positive	11 (11.3%)	13 (16.7%)		22 (14.8%)	2 (7.7%)	
Management							
Thyroidectomy	Unilateral	40 (41.2%)	36 (46.2%)	0.54	62 (41.6%)	14 (53.8%)	0.29
	Total/subtotal	57 (58.8%)	42 (53.8%)		87 (58.4%)	12 (46.2%)	
Neck dissection	Negative	43 (44.3%)	38 (48.7%)	0.65	67 (45%)	14 (53.8%)	0.52
	Positive	54 (55.7%)	40 (51.3%)		82 (55%)	12 (46.2%)	
Radioiodine ablation	Negative	68 (70.1%)	53 (67.9%)	0.87	102 (68.5%)	19 (73.1%)	0.82
	Positive	29 (29.9%)	25 (32.1%)		47 (31.5%)	7 (26.9%)	
External beam radiation therapy	Negative	84 (86.6%)	0 (0%)	**<0.001**	72 (48.3%)	12 (46.2%)	0.83
	Positive	13 (13.4%)	78 (100%)		77 (51.7%)	14 (53.8%)	
Follow-up							
Progression/relapse	Negative	56 (57.7%)	45 (57.7%)	0.99	84 (56.4%)	17 (65.4%)	0.52
	Positive	41 (42.3%)	33 (42.3%)		65 (43.6%)	9 (34.6%)	
Recurrence	Negative	83 (85.6%)	61 (78.2%)	0.24	120 (80.5%)	24 (92.3%)	0.17
	Positive	14 (14.4%)	17 (21.8%)		29 (19.5%)	2 (7.7%)	
Mortality	Survived	93 (95.9%)	74 (94.9%)	0.75	142 (95.3%)	25 (96.2%)	0.84
	Died	4 (4.1%)	4 (5.1%)		7 (4.7%)	1 (3.8%)	

Data are represented as frequency (percentage). Two-sided Chi-square or Fisher’s exact tests were used. Bold values indicate statistical significance at *p* < 0.05.

**Table 2:** Characteristics of cohorts with upregulated miR-145 levels in thyroid tumor tissues.

Patient Characteristics	Levels	Downregulated	Upregulated	*p*-Value
Total number		153	22	
Demographic data				
Age, years	<55 years	105 (68.6%)	14 (63.6%)	0.63
	≥55 years	48 (31.4%)	8 (36.4%)	
Sex	Female	105 (68.6%)	14 (63.6%)	0.63
	Male	48 (31.4%)	8 (36.4%)	
Obesity	Negative	12 (7.8%)	2 (9.1%)	0.69
	Positive	141 (92.2%)	20 (90.9%)	
Smoking	Negative	139 (90.8%)	21 (95.5%)	0.70
	Positive	14 (9.2%)	1 (4.5%)	
Hepatitis C virus	Negative	78 (51%)	21 (95.5%)	**<0.001**
	Positive	75 (49%)	1 (4.5%)	
Hashimoto’s thyroiditis	Negative	131 (85.6%)	18 (81.8%)	0.75
	Positive	22 (14.4%)	4 (18.2%)	
Genomic mutation				
*BRAF*^V600E^ mutation	Negative	90 (58.8%)	7 (31.8%)	**0.022**
	Positive	63 (41.2%)	15 (68.2%)	
RAS gene mutation	Negative	140 (91.5%)	20 (90.9%)	0.92
	Positive	13 (8.5%)	2 (9.1%)	
Pathological data				
Laterality	Unilateral	108 (70.6%)	12 (54.5%)	0.15
	Bilateral	45 (29.4%)	10 (45.5%)	
Focality	Unifocal	129 (84.3%)	16 (72.7%)	0.22
	Multifocal	24 (15.7%)	6 (27.3%)	
T stage	T1a	41 (26.8%)	6 (27.3%)	0.93
	T1b	41 (26.8%)	5 (22.7%)	
	T2	60 (39.2%)	10 (45.5%)	
	T3	7 (4.6%)	1 (4.5%)	
	T4	4 (2.6%)	0 (0%)	
N stage	N0	88 (57.5%)	13 (59.1%)	0.89
	N1	65 (42.5%)	9 (40.9%)	
M stage	M0	130 (85%)	15 (68.2%)	0.06
	M1	23 (15%)	7 (31.8%)	
Pathology stage	Stage Ia	103 (67.3%)	12 (54.5%)	0.51
	Stage II	12 (7.8%)	2 (9.1%)	
	Stage III	11 (7.2%)	1 (4.5%)	
	Stage IVA	9 (5.9%)	2 (9.1%)	
	Stage IVB	3 (2%)	0 (0%)	
	Stage IVC	15 (9.8%)	5 (22.7%)	
Extrathyroidal extension	Negative	121 (79.1%)	16 (72.7%)	0.58
	Positive	32 (20.9%)	6 (27.3%)	
Perineural invasion	Negative	149 (97.4%)	22 (100%)	0.44
	Positive	4 (2.6%)	0 (0%)	
Extranodal extension	Negative	142 (92.8%)	21 (95.5%)	0.65
	Positive	11 (7.2%)	1 (4.5%)	
Lymphovascular invasion	Negative	134 (87.6%)	17 (77.3%)	0.19
	Positive	19 (12.4%)	5 (22.7%)	
Management				
Thyroidectomy	Unilateral	65 (42.5%)	11 (50%)	0.65
	Total/subtotal	88 (57.5%)	11 (50%)	
Neck dissection	Negative	70 (45.8%)	11 (50%)	0.82
	Positive	83 (54.2%)	11 (50%)	
Radioiodine ablation	Negative	104 (68%)	17 (77.3%)	0.47
	Positive	49 (32%)	5 (22.7%)	
External beam radiation therapy	Negative	78 (51%)	6 (27.3%)	**0.042**
	Positive	75 (49%)	16 (72.7%)	
Follow-up				
Progression/relapse	Negative	88 (57.5%)	13 (59.1%)	0.89
	Positive	65 (42.5%)	9 (40.9%)	
Recurrence	Negative	124 (81%)	20 (90.9%)	0.37
	Positive	29 (19%)	2 (9.1%)	
Mortality	Survived	147 (96.1%)	20 (90.9%)	0.26
	Died	6 (3.9%)	2 (9.1%)	

Data are represented as frequency (percentage). Two-sided Chi-square or Fisher’s exact tests were used. Bold values indicate statistical significance at *p* < 0.05.

**Table 3:** Association of MMP9/miR-145 expression with clinical and pathological characteristics of the thyroid tumor (*n* = 175 cases).

Characteristics	Levels	Count	MMP9	miR–145	MMP9/miR–145
Demographic data					
Age, years	<55 years	119	2.89 (1.8–8.2)	0.23 (0.1–0.4)	16.59 (6–51.3)
	≥55 years	56	4.58 (2–10.7)	0.22 (0.1–0.5)	18.16 (9.5–52.4)
Sex	Female	119	2.99 (1.7–8.3)	0.20 (0.1–0.5)	16.86 (7.5–49.6)
	Male	56	4.46 (2.1–10.3)	0.26 (0.1–0.5)	25.05 (6.2–58.5)
Obesity	Negative	14	2.86 (1.7–9.8)	0.33 (0.1–0.8)	10.90 (3.5–30.7)
	Positive	161	3.36 (1.8–8.7)	0.22 (0.1–0.4)	18.65 (7.3–51.7)
Smoking	Negative	160	3.31 (1.8–8.7)	0.22 (0.1–0.4)	16.73 (6.7–52)
	Positive	15	3.93 (2.1–7.5)	0.24 (0.2–0.6)	27.04 (5.4–34.2)
Hepatitis C virus	Negative	99	**2.33 (1.4–5.2)** ***	**0.33 (0.2–0.9)** ***	**8.01 (4.4–16.3)** ***
	Positive	76	**7.53 (2.8–12.2)**	**0.11 (0–0.2)**	**57.87 (37.8–113)**
Hashimoto’s thyroiditis	Negative	149	3.36 (1.8–8.6)	0.23 (0.1–0.4)	18.65 (7.3–52.6)
	Positive	26	2.70 (1.9–9.7)	0.24 (0.1–0.5)	15.15 (4.6–46.2)
Genomic mutation					
*BRAF*^V600E^ mutation	Negative	97	2.99 (1.8–8.9)	0.20 (0.1–0.3)	26.45 (7.7–57.9)
	Positive	78	3.88 (1.8–8.4)	0.26 (0.1–0.8)	13.29 (4.5–47)
RAS gene mutation	Negative	160	3.47 (1.8–8.4)	0.23 (0.1–0.5)	17.24 (6.7–51.1)
	Positive	15	2.89 (1.9–9.4)	0.20 (0.1–0.4)	28.72 (5.6–75)
Pathological data					
Laterality	Unilateral	120	3.23 (1.8–8.6)	0.23 (0.1–0.4)	23.70 (5.9–55.3)
	Bilateral	55	3.68 (1.9–8.7)	0.21 (0.1–0.6)	14.31 (7–41.7)
Focality	Unifocal	145	**2.82 (1.7–8.2)** *	0.23 (0.1–0.4)	16.59 (5.9–51.7)
	Multifocal	30	**5.61 (3.2–11.7)**	0.21 (0.1–0.6)	18.80 (10.4–51)
T stage	T1a	47	**2.36 (1.4–4.5)** **	0.26 (0.1–0.5)	10.03 (4.4–28.7)
	T1b	46	**5.86 (2.8–11.5)**	0.26 (0.1–0.7)	17.24 (8.4–66.4)
	T2	70	**3.78 (1.7–9.3)**	0.20 (0.1–0.3)	27.36 (7.5–56.4)
	T3	8	**2.28 (1.2–7.3)**	0.10 (0–0.3)	26.44 (13.1–48.9)
	T4	4	**5.02 (2.5–16.7)**	0.17 (0.1–0.3)	41.91 (18.1–103.5)
N stage	N0	101	**2.25 (1.4–4.3)** ***	**0.25 (0.1–0.5)** **	**10.85 (4.6–24.6)** ***
	N1	74	**7.84 (3.5–11.8)**	**0.19 (0.1–0.3)**	**47.65 (16.8–102.4)**
M stage	M0	145	**2.72 (1.6–7.9)** ***	0.21 (0.1–0.4)	16.59 (5.9–50.5)
	M1	30	**7.88 (3.8–11.7)**	0.28 (0.1–1)	27.62 (9.7–56.7)
Pathology Stage	Stage Ia	11	**2.61 (1.7–7.8)** **	0.23 (0.1–0.4)	15.23 (5.6–47.2)
	Stage II	3	**7.04 (3.6–12.6)**	0.21 (0.1–0.5)	43.30 (14.1–125.3)
	Stage III	20	**2.54 (1.6–4.6)**	0.16 (0–0.4)	22.30 (4.9–51.7)
	Stage IVA	115	**2.19 (1.2–7.9)**	0.10 (0–0.2)	18.65 (10.1–80.6)
	Stage IVB	14	**17.97 (1.9–0)**	0.34 (0.1–0)	53.06 (15.1–0)
	Stage IVC	12	**8.05 (4.2–12.1)**	0.34 (0.2–1.1)	17.27 (9.7–47.2)
Extrathyroidal extension	Negative	137	3.07 (1.8–8.3)	0.22 (0.1–0.4)	17.67 (6.7–51.7)
	Positive	38	4.13 (1.8–10.6)	0.24 (0.1–0.9)	16.46 (7.4–50)
Perineural invasion	Negative	171	3.36 (1.8–8.7)	0.23 (0.1–0.5)	16.86 (6.6–50.4)
	Positive	4	2.85 (2.4–9.9)	0.15 (0–0.3)	49.84 (16.4–73.2)
Extranodal extension	Negative	163	3.22 (1.8–8.4)	0.23 (0.1–0.5)	16.59 (6.6–49.6)
	Positive	12	6.24 (2.1–12.2)	0.15 (0–0.3)	42.62 (19.3–64.7)
Lymphovascular invasion	Negative	151	2.89 (1.8–8.2)	0.23 (0.1–0.5)	16.59 (6–49.6)
	Positive	24	6.85 (3.3–11.1)	0.17 (0.1–0.8)	33.00 (10.5–64.7)
Follow-up					
Progression/relapse	Negative	101	**2.12 (1.4–3.9)** ***	**0.25 (0.1–0.5)** *	**10.28 (4.6–24.2)** ***
	Positive	74	**8.05 (3.9–12.4)**	**0.21 (0.1–0.3)**	**48.57 (17.5–106.6)**
Recurrence	Negative	144	**2.83 (1.6–7.8)** **	**0.25 (0.1–0.5)** **	**13.64 (5.7–37.3)** ***
	Positive	31	**7.90 (3.2–13.1)**	**0.09 (0–0.2)**	**83.78 (34.2–122.4)**
Mortality	Survived	167	3.27 (1.8–8.4)	0.23 (0.1–0.5)	17.62 (6.7–52.2)
	Died	8	3.91 (1.6–10.3)	0.24 (0.1–0.9)	22.66 (5.5–44.5)

Data are presented as median and interquartile range (IQR). RAS genes included NRAS, HRAS, and KRAS genes. Values of relative expression levels are shown in tumor tissues compared to paired adjacent non-tumor tissues. Comparison between groups was performed using Mann–Whitney U and Kruskal–Wallis 1-way ANOVA tests. Bold values indicate statistical significance at *p* < 0.05 and are represented with asterisks (*). * *p* < 0.05, ** *p* < 0.01, *** *p* < 0.001.

**Table 4:** Performance of MMP9/miR-145 ratio on predicting poor prognosis.

Comparison	Cohort	Control	Cases	AUC	Sensitivity	Specificity	*p-*Value
Nodal metastasis vs. none	Overall	101	74	0.791	73.0	72.3	<0.001
	*BRAF*−	54	43	0.854	86.0	70.4	<0.001
	*BRAF*+	47	31	0.722	54.8	74.5	<0.001
	HT−	83	66	0.798	72.7	72.3	<0.001
	HT+	18	8	0.757	75.0	72.2	0.040
Progression/relapse vs. none	Overall	101	74	0.812	74.3	73.3	<0.001
	*BRAF*−	56	41	0.892	90.2	71.4	<0.001
	*BRAF*+	45	33	0.733	54.5	75.6	<0.001
	HT−	84	65	0.797	72.3	71.4	<0.001
	HT+	17	9	0.895	88.9	82.4	0.001
Recurrence vs. none	Overall	61	17	0.807	70.6	72.1	<0.001
	*BRAF*−	83	14	0.827	92.9	51.8	<0.001
	*BRAF*+	61	17	0.817	70.6	72.1	<0.001
	HT−	120	29	0.814	82.8	60.8	<0.001
	HT+	24	2	0.750	50.0	58.3	0.25

*BRAF*: *BRAF*^V600E^ mutation; HT: Hashimoto’s thyroiditis; AUC: area under the curve. Receiver operator characteristic curve analysis was performed. Statistical significance was set at *p*-value < 0.05.

**Table 5:** Association between MMP9/miR-145 ratio and clinical outcomes in PTC patients (*n* = 86).

Characteristics		*n* (%)	Tissue	Pre-Plasma	Post-Plasma	*p*-Value (Pre vs. Tissue)	*p*-Value (Post vs. Pre)
PTC patients	Overall	86 (100)	17.6 (5.3–64.0)	2.39 (0.61–9.71)	0.74 (0.24–2.25)	<0.001	0.011
LNM	Negative	44 (52.2)	7.8 (3.9–18.1)	1.34 (0.36–4.2)	0.91 (0.22–2.60)	<0.001	0.41
	Positive	42 (48.8)	56.9 (21.4–116)	6.65 (1.98–11.6)	0.57 (0.24–1.76)	<0.001	0.026
Extrathyroidal extension	Negative	67 (77.9)	23.1 (6.0–63.58)	0.74 (0.22–1.78)	2.15 (0.42–4.96)	<0.001	0.013
	Positive	19 (22.1)	2.61 (0.75–10.6)	16.3 (4.53–65.5)	0.70 (0.43–4.40)	<0.001	1.0
Progression/relapse	Negative	47 (54.7)	7.7 (3.97–18.65)	0.90 (0.22–2.68)	7.81 (2.61–13.4)	0.028	0.37
	Positive	39 (45.3)	1.32 (0.37–3.40)	59.2 (27.0–119)	0.6 (0.25–1.77)	<0.001	<0.001
Recurrence	Negative	69 (80.2)	13.27 (4.6–43.0)	1.81 (0.52–7.62)	0.83 (0.26–2.27)	<0.001	0.07
	Positive	17 (19.8)	162.2 (93.9–182)	13.9 (8.27–17.8)	2.26 (0.52–5.79)	<0.001	0.18
Died	Negative	82 (95.3)	17.4 (5.27–64.1)	0.73 (0.24–2.25)	3.98 (1.01–9.99)	<0.001	0.009
	Positive	4 (4.7)	2.17 (0.62–9.72)	6.5 (5.8–14.1)	1.03 (0.35–11.2)	0.10	1.0

The analysis was performed using the related-samples Friedman’s two-way analysis of variance by ranks test. All significant values have been adjusted by the Bonferroni correction for multiple tests. Statistical significance was set at *p*-value < 0.05. *n* (%): number (percentage), LNM: lymph node metastasis.

**Table 6:** Association of *MMP9* gene and protein expression with clinicopathological characteristics.

Ref.	Author	Year	Country	Source	Type	PTC	LNM	TNM	Size	M1	ETE	SPRD
[61]	Roncevic	2022	Serbia	Tissue	Protein/RNA	50		•			•	
[78]	EH Kusumastuti	2021	Indonesia	Tissue	Protein	43	•					
[62]	Liu Xincheng	2020	China	Tissue	Protein	104	•	•				
[73]	Dobrescu R	2020	Romania	Serum	Protein	97						•
[63]	Li Na	2019	China	Tissue	Protein	40	•					
[64]	Roncevic J	2019	Serbia	Tissue	Protein/RNA	105	•	•	•		•	
[77]	XingKai Liu	2019	China	Tissue	Protein	112	•	•	•			•
[65]	Dahai Xu	2019	China	Serum	Protein	182	•	•	•	•	•	•
[83]	Boris Bumber	2020	Croatia	Tissue	Protein	159	•					
[74]	Zhang	2019	China	Serum	Protein	57	•	•	•			
[84]	Maryam Zarkesh	2018	Iran	Tissue	Protein/RNA	60		•				
[62]	Chen Suqin	2018	China	Tissue	Protein	200	•	•	•			
[66]	Wei Tian	2017	China	Tissue	Protein	88		•	•			
[21]	Ilona Marecko	2014	Serbia	Tissue	Protein	120	•				•	
[67]	Ni Wang	2014	China	Tissue	Protein	83	•	•	•			
[68]	Yang Lei	2014	China	Tissue	Protein	72	•	•				
[69]	Meng Xian-Ying	2014	China	Tissue	Protein	66	•	•	•			
[62]	Liu Zhen	2014	China	Tissue	Protein	164	•	•	•			
[67]	Ni Wang	2013	China	Tissue	Protein/RNA	83	•		•			
[70]	Huang Pei	2013	China	Tissue	Protein	58	•					
[67]	Xianying Meng	2012	China	Tissue	Protein/RNA	66	•		•			
[62]	Dong Yuhong	2012	China	Tissue	Protein	168	•	•				
[62]	Wang Zhaohui	2012	China	Tissue	Protein	56	•	•				
[71]	Shi Jianhua	2010	China	Tissue	Protein	157	•					
[62]	Zhang Jie	2010	China	Tissue	Protein	74	•					
[79]	Buergy	2009	Germany	Tissue	Protein	67						•
[75]	Shih Lin	2003	Taiwan	Plasma	Protein	30	•	•	•	•	•	

Immunohistochemistry, ELISA, and gelatin zymography were employed to assess proteins. PCR-RFLP was used to quantify the mRNA. Some articles were exclusively in Chinese journals and were not available as full texts, and data were retrieved from a recent meta-analysis of Wen et al. [62]. PTC: Papillary thyroid cancer. LNM: lymph node metastasis. TNM: tumor nodal metastasis staging system. T size: tumor size. M1: distant metastasis. ETE: extrathyroidal extension. SPRD: structurally persistent/recurrent disease.

## Data Availability

Data are available from the corresponding author upon reasonable request and obtaining the approval of the “Office of Technology Transfer and Intellectual Property Development, Tulane University, USA”. The sources of the in silico data analysis sections are available in public repositories through the links provided in the manuscript.

## Supplementary Materials

The following supporting information can be downloaded at https://www.mdpi.com/article/10.3390/biomedicines11112953/s1, Table S1: Antibodies used in immunofluorescence analysis.
