# Supplementary material for: Matrix Metalloproteinase 9/microRNA-145 Ratio: Bridging Genomic and Immunological Variabilities in Thyroid Cancer
Source: Biomedicines. 2023 Nov 1;11(11):2953. doi: 10.3390/biomedicines11112953 (PMC10669161; doi:10.3390/biomedicines11112953)
Supplement: Supplementary file 1 [file biomedicines-11-02953-s001.zip › biomedicines-2645745-supplementary.pdf]

Supplementary Table S1. Antibodies used in immunofluorescence analysis.

Primary antibody	Species	Company	Catalog#	Dilution	Secondary antibody
MMP9	Goat	R&D	AF911	1:100	Alexa Fluor 488 Donkey anti-Goat
CD45	Mouse IgG1	Abcam	Ab781	1:50	Alexa Fluor 555 Donkey anti-mouse
DAPI		Invitrogen	D1306	1:20,000	
